# Broadly resistant HIV-1 against CD4-binding site neutralizing antibodies

**DOI:** 10.1371/journal.ppat.1007819

**Published:** 2019-06-13

**Authors:** Panpan Zhou, Han Wang, Mengqi Fang, Yangyang Li, Hua Wang, Shasha Shi, Zihao Li, Jiapeng Wu, Xiaoxu Han, Xuanling Shi, Hong Shang, Tongqing Zhou, Linqi Zhang

**Affiliations:** 1 Comprehensive AIDS Research Center, Collaborative Innovation Center for Diagnosis and Treatment of Infectious Diseases, Beijing Advanced Innovation Center for Structural Biology, Department of Basic Medical Sciences, School of Medicine, Tsinghua University, Beijing, China; 2 Key Laboratory of AIDS Immunology of the Ministry of Health, Department of Laboratory Medicine, No. 1 Hospital of China Medical University, Shenyang, China; 3 Vaccine Research Center, National Institute of Allergy and Infectious Diseases and National Institutes of Health, Bethesda, MD, United States of America; University of Zurich, SWITZERLAND

## Abstract

Recently identified broadly neutralizing antibodies (bnAbs) show great potential for clinical interventions against HIV-1 infection. However, resistant strains may impose substantial challenges. Here, we report on the identification and characterization of a panel of HIV-1 strains with broad and potent resistance against a large number of bnAbs, particularly those targeting the CD4-binding site (CD4bs). Site-directed mutagenesis revealed that several key epitope mutations facilitate resistance and are located in the inner domain, loop D, and β23/loop V5/β24 of HIV-1 gp120. The resistance is largely correlated with binding affinity of antibodies to the envelope trimers expressed on the cell surface. Our results therefore demonstrate the existence of broadly resistant HIV-1 strains against CD4bs neutralizing antibodies. Treatment strategies based on the CD4bs bnAbs must overcome such resistance to achieve optimal clinical outcomes.

## Author summary

Recently identified broadly neutralizing antibodies (bnAbs) show great potential for clinical interventions against HIV-1 infection. Among the bnAbs isolated to date, those targeting the CD4bs are the most abundant and thoroughly studied as they disrupt the crucial step of viral interaction with the cellular receptor molecule CD4. Despite the superior potency and breadth of these CD4bs bnAbs, each fails to neutralize a small but significant portion of pseudotyped virus panels. Here, we report on the identification and characterization of a panel of HIV-1 strains with broad and potent resistance against a large number of bnAbs, particularly those targeting the CD4bs. Resistance is largely attributed to mutated residues within the epitopes or steric hindrance imposed by the bulky side-chain or glycan shield of the mutated residues, and is largely correlated with reduced binding avidity of the antibody to the quaternary trimeric envelope protein expressed on the surface of the transfected cells. Treatment strategies based on the CD4bs bnAbs therefore must overcome such resistance to achieve optimal clinical outcomes.

## Introduction

Recent scientific advances have identified a growing number of broadly neutralizing antibodies (bnAbs) against human immunodeficiency virus type I (HIV-1), providing promising candidates for HIV-1 prevention and treatment [1–5]. Compared with bnAbs isolated in earlier studies, these bnAbs demonstrated broader and more potent activity against global HIV-1 panels and displayed impressive safety and efficacy profiles for therapeutic applications in a number of animal models and human clinical trials[1, 6–11]. These results also offer the opportunity to tailor these antibodies and maximize their prevention and treatment potential. Broadly speaking, these bnAbs recognize six major “vulnerable sites” on the HIV-1 envelope glycoprotein gp160 (gp120 and gp41), which are (1) the CD4-binding site (CD4bs), (2) the V1V2 apex, (3) the V3 glycan, (4) the fusion peptide (FP), (5) subunit interface and (6) membrane proximal external region (MPER) of gp41[1–5]. These bnAbs neutralize HIV-1 by blocking viral entry into the target cells, although additional effector functions are likely at play, particularly in the context of *in vivo* infection and clinical application[1, 12–16].

Among the bnAbs isolated to date, those targeting the CD4bs are the most abundant and thoroughly studied as they disrupt the first and crucial step of viral interaction with the cellular receptor CD4 [3,4,17,18]. In this step, the trimeric envelope glycoprotein, composed of gp120 and gp41, binds with the target cell through the receptor CD4, with either CCR5 or CXCR4 as co-receptor[19–21]. This triggers a cascade of conformational changes that fuses the viral envelope with the target membrane and releases the viral genome into the cell [22, 23].More than two dozen CD4bs bnAbs have been isolated to date. This number is likely to grow with the advent of more specific and high-throughput techniques for isolating antibodies.For instance, the prototype VRC01 and its close relative VRC03, which effectively neutralize a panel of diverse pseudotyped viruses,were isolated from a clade-B infected individual [24–26].The more recently isolated CD4bs bnAbs, such as 3BNC117, N6, N49P7, 3BNC60, VRC-PG04, VRC-PG20, NIH45-46, VRC-CH31, 12A12, CH103, 8ANC131, VRC13, and VRC16,have shown similar or even enhanced potency and breadth compared with VRC01[1–5, 27, 28].The CD4bs bnAbs are classified into two groups based on their mode of recognition and heavy chain characteristics, such as the VRC01 class(3BNC117, N6, N49P7, 3BNC60, VRC-PG04, VRC-PG20, NIH45-46, VRC-CH31 and 12A12) and non-VRC01 classes (CH103, 8ANC131, VRC13 and VRC16)[17, 18]. Structurally, these bnAbs recognize residues within the inner domain, the Loop D, the CD4 binding loop, and the β23/loop V5/β24 region [17, 18, 29–31].The V1V2 and V3 loop also impact recognition,particularly in the context of the quaternary trimeric envelope protein[32–36].The primary mechanism of neutralization mimics and competes with the CD4 receptorto bind HIV-1 gp120, thereby preventing bound trimers from transitioning to the subsequent steps required for membrane fusion, although evidence indicates that there are differences in the fine details of this process[17, 18, 35, 36].

Despite the superior potency and breadth of these CD4bs bnAbs, each fails to neutralize a small but significant portion of pseudotyped virus panels[17, 25, 27, 28, 37, 38].For instance, VRC01 is unable to neutralize about 10% of tested viruses[25], and resistant strains have been isolated from patients infected with subtype B, subtype C, CRF07_BC, and CRF08_BC[29, 39–42]. Furthermore, reports show thatVRC01-, 3BNC117- and N6-resistant strains have emerged in both animal models and human clinical trials[11, 15, 43–47], indicating that resistant virus strains occur naturally and are selected for during antibody treatment.As more clinical trials on bnAbs are underway, more resistant viruses are expected to emerge. Identification and characterization of these resistant strains is paramount for understanding the molecular basis of their resistance and ultimately developing strategies to effectively control them.

This study reports on the identification and characterization of a panel of HIV-1 strains with broad and potent resistance against a large number of bnAbs, particularly those targeting the CD4bs. We hereafter referred these resistant clones as “Resistant panel to CD4bs bnAbs”. The clones within the panel were derived from envelope sequences that we collected from acutely and chronically infected patients, as well as from earlier literature reports[39, 48–50]. Systemic analysis of the resistant clones revealed the critical residues within the CD4bs of HIV-1 gp120,as well as the molecular features that confer broad resistance.Resistance was either due to the mutated residues themselves or steric hindrance imposed by the bulky side-chain or glycan shield of the mutated residues. The level of resistance is largely correlated with reduced binding avidity of the antibody to the quaternary trimeric envelope protein expressed on the surface of the transfected cells. Our results highlight the existence of HIV-1 strains broadly resistant against CD4bs bnAbs in the infected population.Treatment strategies based on the CD4bs bnAbs would need to overcome such resistance to achieve optimal clinical outcomes.

## Results

### Identification of broadly resistant HIV-1 against CD4bs neutralizing antibodies

We previously characterized a large number of full-length HIV-1envelope sequences from infected individuals in China. These are widely distributed among the three major genetic clusters: subtype CRF01_AE, subtype B', and subtype C/CRF07_BC/CRF08_BC/B'C [39].When pseudoviruses bearing these Env were exposed to subtype-specific plasma pools and bnAbs, we observed substantial differences in neutralization sensitivity. Many clones were resistant to one or more bnAbs,including those known to have high potency and breadth against diverse HIV-1strains from outside of China.Some have been selected for the Global Panel of HIV-1 Env Reference Clones under the NIH-AIDS Reagent Program (Cat #12670)based on their unique genotypic and phenotypic features[48].As more bnAbs have since been identified,we sought to reevaluate the resistance of these clones against a larger array of antibodies, with a particular focus on those targeting the CD4bs (Table 1).Six clones derived from the Chinese panels (CNE6, CNE23,CNE63, CNE64, CNE66 and BJOX2000) showed variable but broad resistance against the most potent CD4bs bnAbs in both the VRC01 and non-VRC01 class families (Table 1). For instance, CNE6 and CNE66 were resistant to 15 of the 16 CD4bs bnAbs at a 50% inhibitory concentration (IC_50_) of less than 50 μg/ml. CNE63 and its cognate CNE64,derived from the same patient,were resistant to more than or equal to 13 of 16 CD4bs bnAbs. CNE23 and BJOX2000 evaded 10 of the 16 CD4bs bnAbs tested.The resistance level rose when neutralization was more stringently defined at an IC_80_ of less than 50 μg/ml (Table 1).Broadly resistant clones were also identified in the non-Chinese panels(Table 1, right panel)[29, 30, 50]three of which (BL01, TV1.29 and TZA125.17)were verified and selected here for more in-depth mutational analyses. BL01 demonstrated resistance to all 16 CD4bs tested, while TV1.29 and TZA125.17 were resistant to 15 and 14respectively(Table 1, left panel). Furthermore, some clones were equally resistant to bnAbs targeting other Env sites,such as V1V2, V3-glycan, subunit interface, fusion peptide and MPER (Table 1).It should be noted that these clones were derived from patients with both acute (BJOX2000) and chronic (CNE6, CNE23, CNE63, CNE64, CNE66, BL01, TV1.29 and TZA125.17) infection, and none of them received any intervention related to bnAbs.Notably, among the previously published non-Chinese clones, all except X2088.c9 were isolated during acute or early infection (Table 1, right panel). These findings indicate that the broad resistance was not only natural occurring, but was also transmissible and the capable of replicating in newly infected patients.

**Table 1:** Background information and neutralization sensitivity of broadly resistant HIV-1 strains.

	**IC**_**50**_**/IC**_**80**_**(μg/ml)**					**NC**		**Senstive studied**				**Resistant studied**																		**Resisitant reported**																			
				**Virus ID**		AMLV		SF162		JRFL		CNE6		CNE23		CNE63		CNE64		CNE66		BJOX2000		BL01		TV1.29		TZA125.17		T278-50		242–14		T250-4		HO86.8		DU422.01		X2088.c9		6322.V4.C1		6471.V1.C16		6631.V3.C10		620345.c1	
		<0.1		**GenBank ID**		M33469.1		EU123924.1		U63632.1		HM215423.1		HM215408.1		HQ699978.1		HQ699979.1		HQ699981.1		HM215364.1		AY124970.1		EU855132.1		JQ362423.1		EU513198.1		EU513188.1		EU513189.1		EF210732.1		DQ411854.1		EU885764.1		HM215326.1		HM215328.1		HM215335.1		JQ362422.1	
		0.1–1		**Clade**		NA		B		B		B		BC		B		B		C		BC		B		C		C		AG		AG		AG		B		C		G		C		C		C		AE	
		1–10		**Country of origin**		NA		America		America		China		China		China		China		China		China		America		South Africa		Tanzania		Cameroon		Cameroon		Cameroon		Peru		South Africa		Spain		Tanzania		Tanzania		Tanzania		Thailand	
		10–50		**Stage of infection**		NA		early		chronic		chronic		chronic		chronic		chronic		chronic		acute		chronic		chronic		chronic		early		early		early		early		early		chronic		early		early		early		acute	
		>50		**Mode of transmission**		NA		sexual		sexual		IDU		IDU		Blood		Blood		IDU		IDU		NA		sexual		NA		NA		NA		NA		sexual		sexual		sexual		sexual		sexual		sexual		sexual	
				**Year of collection**		NA		NA		1986		2006		2007		2004		2004		2007		2007		NA		1998		2001		2005		2004		2004		NA		1998		2006		2005		2003		2004		2005	
				**Coreceptor usage**		NA		R5		R5		R5		R5		R5		R5		R5		R5		R5/X4		R5		R5		NA		NA		NA		NA		R5		R5		NA		NA		NA		NA	
**Epitope**			**bnAb ID**	**Neutralization breadth**		IC_50_	IC_80_	IC_50_	IC_80_	IC_50_	IC_80_	IC_50_	IC_80_	IC_50_	IC_80_	IC_50_	IC_80_	IC_50_	IC_80_	IC_50_	IC_80_	IC_50_	IC_80_	IC_50_	IC_80_	IC_50_	IC_80_	IC_50_	IC_80_	IC_50_	IC_80_	IC_50_	IC_80_	IC_50_	IC_80_	IC_50_	IC_80_	IC_50_	IC_80_	IC_50_	IC_80_	IC_50_	IC_80_	IC_50_	IC_80_	IC_50_	IC_80_	IC_50_	IC_80_
CD4bs	VRC01 class		VRC01	656/783 (84%)	1/19(5%)	>50	>50	0.05	0.22	3.98	22.57	>50	>50	42.00	>50	>50	>50	>50	>50	>50	>50	>50	>50	>50	>50	>50	>50	>50	>50	>50	>50	>50	>50	>50	>50	>50	>50	>50	>50	>50	>50	>50	>50	>50	>50	>50	>50	>50	>50
			3BNC117	357/455 (78%)	0/19(0%)	>50	>50	0.01	0.02	0.05	0.16	>50	>50	>50	>50	>50	>50	>50	>50	>50	>50	>50	>50	>50	>50	>50	>50	>50	>50	>50	>50	>50	>50	>50	>50	>50	>50	>50	>50	>50	>50	>50	>50	>50	>50	>50	>50	>50	>50
			N6	347/356 (97%)	14/19(74%)	>50	>50	0.02	0.04	0.01	0.04	>50	>50	17.00	>50	17.00	>50	5.80	16.00	0.06	0.25	0.81	2.38	>50	>50	>50	>50	0.23	2.30	>50	>50	0.26	NA	0.01	NA	0.45	NA	0.02	NA	0.05	NA	0.03	NA	>50	>50	0.07	NA	0.34	NA
			N49P7	118/118(100%)	9/14(64%)	>50	>50	0.07	0.32	0.03	0.18	>50	>50	34.55	>50	>50	>50	36.06	>50	>50	>50	9.87	30.62	>50	>50	>50	>50	6.49	48.13	NA	NA	NA	NA	NA	NA	NA	NA	NA	NA	NA	NA	NA	NA	NA	NA	NA	NA	NA	NA
			3BNC60	41/45 (91%)	0/13(0%)	>50	>50	0.01	0.02	0.04	0.14	>50	>50	>50	>50	>50	>50	>50	>50	>50	>50	>50	>50	>50	>50	>50	>50	>50	>50	>50	>50	NA	NA	>50	>50	>50	>50	NA	NA	NA	NA	NA	NA	NA	NA	NA	NA	>50	>50
			VRC-PG04	354/441 (80%)	2/19(11%)	>50	>50	1.28	6.88	3.26	23.93	>50	>50	49.00	>50	>50	>50	>50	>50	>50	>50	3.50	44.00	>50	>50	>50	>50	>50	>50	>50	>50	>50	>50	>50	>50	>50	>50	>50	>50	>50	>50	>50	>50	>50	>50	>50	>50	>50	>50
			VRC-PG20	141/183 (77%)	2/19(11%)	>50	>50	12.88	44.61	6.28	23.28	>50	>50	>50	>50	>50	>50	>50	>50	>50	>50	0.60	13.00	>50	>50	>50	>50	>50	>50	>50	>50	>50	>50	0.07	0.74	>50	>50	>50	>50	>50	>50	>50	>50	>50	>50	>50	>50	>50	>50
			b12	306/866 (35%)	2/19(11%)	>50	>50	0.08	0.33	0.06	0.19	>50	>50	0.45	14.00	>50	>50	>50	>50	>50	>50	>50	>50	>50	>50	>50	>50	>50	>50	>50	>50	>50	>50	>50	>50	>50	>50	0.46	1.80	>50	>50	>50	>50	>50	>50	>50	>50	>50	>50
			NIH45-46	227/271 (84%)	1/19(5%)	>50	>50	1.58	5.48	10.30	30.81	>50	>50	5.50	45.00	>50	>50	>50	>50	>50	>50	>50	>50	>50	>50	>50	>50	>50	>50	>50	>50	>50	>50	>50	>50	>50	>50	>50	>50	>50	>50	>50	>50	>50	>50	>50	>50	>50	>50
			VRC-CH31	218/270 (81%)	0/19(0%)	>50	>50	49.62	>50	0.39	1.79	>50	>50	>50	>50	>50	>50	>50	>50	>50	>50	>50	>50	>50	>50	>50	>50	>50	>50	>50	>50	>50	>50	>50	>50	>50	>50	>50	>50	>50	>50	>50	>50	>50	>50	>50	>50	>50	>50
			VRC03	182/334 (54%)	0/19(0%)	>50	>50	0.09	0.34	0.74	4.56	>50	>50	>50	>50	>50	>50	>50	>50	>50	>50	>50	>50	>50	>50	>50	>50	>50	>50	>50	>50	>50	>50	>50	>50	>50	>50	>50	>50	>50	>50	>50	>50	>50	>50	>50	>50	>50	>50
			12A12	166/178 (93%)	1/15(7%)	>50	>50	0.02	0.47	3.95	30.25	>50	>50	>50	>50	>50	>50	>50	>50	>50	>50	34.00	>50	>50	>50	>50	>50	>50	>50	>50	>50	NA	NA	>50	>50	>50	>50	>50	>50	>50	>50	NA	NA	NA	NA	NA	NA	>50	>50
	non-VRC01 classes		8ANC131	141/183 (77%)	2/19(11%)	>50	>50	1.00	3.33	0.80	0.32	>50	>50	>50	>50	>50	>50	>50	>50	>50	>50	18.13	>50	>50	>50	>50	>50	>50	>50	>50	>50	>50	>50	>50	>50	>50	>50	>50	>50	0.63	NA	>50	>50	>50	>50	>50	>50	>50	>50
			CH103	167/196 (85%)	2/19(11%)	>50	>50	0.09	0.22	0.10	0.32	>50	>50	>50	>50	>50	>50	>50	>50	>50	>50	>50	>50	>50	>50	>50	>50	>50	>50	>50	>50	7.70	NA	>50	>50	>50	>50	>50	>50	>50	>50	>50	>50	>50	>50	>50	>50	0.05	NA
			VRC13	270/348 (78%)	8/19(42%)	>50	>50	0.66	3.71	0.13	0.41	1.20	>50	>50	>50	6.70	44.00	0.02	0.46	>50	>50	>50	>50	>50	>50	0.06	0.87	>50	>50	>50	>50	0.18	NA	0.38	NA	>50	>50	>50	>50	>50	>50	3.02	>50	>50	>50	>50	>50	0.01	NA
			VRC16	101/175 (58%)	3/19(16%)	>50	>50	0.03	0.09	0.03	0.11	>50	>50	>50	>50	>50	>50	>50	>50	>50	>50	>50	>50	>50	>50	>50	>50	>50	>50	>50	>50	>50	>50	9.00	NA	0.02	NA	>50	>50	15.00	NA	>50	>50	>50	>50	>50	>50	>50	>50
	**Overall average**			3792/5202(73%)	47/289(16%)	0/16(0%)		16/16(100%)		16/16(100%)		1/16(6%)		6/16(38%)		2/16(13%)		3/16(19%)		1/16(6%)		6/16(38%)		0/16(0%)		1/16(6%)		2/16(13%)		1/16(6%)		3/13(23%)		5/16(31%)		2/15(13%)		3/15(20%)		4/15(27%)		2/13(15%)		0/13(0%)		1/13(8%)		4/16(25%)	
non-CD4bs	V1V2		PG9	548/734 (75%)	14/19(74%)	>50	>50	>50	>50	>50	>50	0.65	1.30	7.40	>50	1.61	13.84	0.03	0.13	0.06	0.43	0.34	7.10	>50	>50	0.01	0.05	0.53	1.80	0.91	6.10	0.03	0.11	0.002	0.01	0.01	0.13	0.30	16.00	>50	>50	>50	>50	>50	>50	>50	>50	1.10	>50
			PGT145	437/625 (70%)	12/19(63%)	>50	>50	>50	>50	33.00	>50	>50	>50	0.21	1.60	>50	>50	>50	>50	0.01	0.05	0.35	9.80	>50	>50	0.90	16.00	0.05	0.18	10.00	>50	0.03	1.36	0.0004	0.03	0.002	0.03	23.00	>50	>50	>50	0.12	2.47	>50	>50	>50	>50	0.06	2.00
			CH01	127/242 (52%)	5/19(26%)	>50	>50	>50	>50	>50	>50	>50	>50	>50	>50	>50	>50	>50	>50	6.10	>50	>50	>50	>50	>50	6.80	>50	>50	>50	1.80	8.50	>50	>50	0.04	0.28	0.19	>50	>50	>50	>50	>50	>50	>50	>50	>50	>50	>50	>50	>50
	Glycan-V3		10–1074	267/420 (64%)	12/19(63%)	>50	>50	0.19	0.65	0.72	3.90	2.30	8.80	>50	>50	2.36	5.55	0.04	0.16	>50	>50	1.20	2.50	>50	>50	36.00	>50	17.34	>50	0.70	4.70	>50	>50	0.002	0.001	>50	>50	0.05	0.11	0.003	0.01	>50	>50	2.20	15.00	0.34	1.50	>50	>50
			PGT128	389/636 (61%)	12/19(63%)	>50	>50	0.001	0.004	0.01	0.01	0.04	1.50	2.30	6.30	0.07	0.25	0.01	0.03	36.67	>50	0.20	0.78	>50	>50	1.00	1.70	4.00	22.00	0.01	0.19	>50	>50	0.002	0.01	>50	>50	0.03	0.11	>50	>50	>50	>50	>50	>50	9.50	>50	>50	>50
			PGT121	408/638 (64%)	8/19(42%)	>50	>50	0.005	0.01	0.02	0.07	0.61	2.90	>50	>50	0.03	0.16	0.22	1.10	>50	>50	0.06	0.32	>50	>50	1.60	9.60	>50	>50	>50	>50	>50	>50	0.003	0.01	>50	>50	0.02	0.08	0.01	0.02	>50	>50	>50	>50	>50	>50	>50	>50
	CD4i		17b	20/221 (9%)	0/19(0%)	>50	>50	1.30	>50	>50	>50	>50	>50	>50	>50	>50	>50	>50	>50	>50	>50	>50	>50	>50	>50	>50	>50	>50	>50	>50	>50	>50	>50	>50	>50	>50	>50	>50	>50	>50	>50	>50	>50	>50	>50	>50	>50	>50	>50
	Subunit interface		35O22	147/276 (53%)	3/19(16%)	>50	>50	>50	>50	0.002	>50	>50	>50	>50	>50	>50	>50	>50	>50	0.001	>50	>50	>50	0.10	2.70	>50	>50	>50	>50	2.27	>50	>50	>50	>50	>50	>50	>50	>50	>50	>50	>50	>50	>50	>50	>50	>50	>50	>50	>50
	Fusion peptide		PGT151	189/261 (72%)	13/19(68%)	>50	>50	0.01	0.11	0.01	0.03	0.002	0.03	0.01	0.03	>50	>50	>50	>50	0.001	0.10	0.004	0.02	0.02	0.09	0.01	0.02	0.28	16.00	0.003	0.07	0.001	0.02	0.001	0.003	5.80	>50	0.40	>50	>50	>50	>50	>50	>50	>50	0.002	0.01	>50	>50
			ACS202	39/87 (45%)	2/14(14%)	>50	>50	0.30	33.05	0.09	0.46	>50	>50	>50	>50	>50	>50	>50	>50	>50	>50	>50	>50	>50	>50	>50	>50	>50	>50	>50	>50	NA	NA	>50	>50	NA	NA	0.07	4.10	>50	>50	NA	NA	NA	NA	NA	NA	0.20	2.17
			VRC34.01	101/208 (49%)	8/19(42%)	>50	>50	11.00	>50	1.01	13.00	>50	>50	>50	>50	>50	>50	>50	>50	>50	>50	0.23	1.40	0.44	>50	0.37	10.34	>50	>50	0.04	0.16	0.18	0.64	0.16	0.67	0.43	3.20	1.10	>50	>50	>50	>50	>50	>50	>50	>50	>50	>50	>50
	MPER		2F5	382/802 (48%)	10/19(53%)	>50	>50	1.90	18.24	0.12	2.10	0.56	19.00	>50	>50	7.34	17.34	0.23	4.80	>50	>50	>50	>50	13.14	27.20	0.88	8.70	>50	>50	4.30	34.00	1.10	5.70	2.90	13.00	0.05	2.30	>50	>50	>50	>50	>50	>50	>50	>50	>50	>50	0.46	7.60
			4E10	732/801 (91%)	16/19(84%)	>50	>50	1.49	27.43	0.16	2.00	0.15	11.00	0.31	3.70	1.99	27.86	0.18	2.00	0.01	0.28	2.00	15.00	6.90	26.00	0.58	5.00	1.73	11.69	3.20	30.00	>50	>50	0.36	5.10	0.34	9.40	1.70	12.00	>50	>50	7.95	40.70	23.00	>50	>50	>50	0.47	9.10
			10E8	418/439 (95%)	18/19(95%)	>50	>50	0.05	0.71	0.05	0.38	0.52	5.00	0.39	2.20	0.63	2.47	0.05	0.63	0.01	0.25	7.20	15.00	4.10	8.70	0.44	1.60	3.60	15.00	0.36	3.00	1.40	6.20	0.66	4.30	0.10	2.40	1.10	2.50	>50	>50	1.80	10.60	4.90	19.00	0.64	3.10	0.75	5.50
	**Overall average**			4204/6390(66%)	133/261(51%)	0/14(0%)		10/14(71%)		11/14(79%)		8/14(57%)		6/14(43%)		7/14(50%)		7/14(50%)		8/14(57%)		9/14(64%)		6/14(43%)		11/14(79%)		7/14(50%)		11/14(79%)		6/13(46%)		11/14(79%)		8/13(62%)		10/14(71%)		2/14(14%)		3/13(23%)		3/13(23%)		4/13(31%)		6/14(43%)	

In the first column, the “Neutralization breadth” compares the percentages of viruses neutralized in Table 1 to that of large panels from the database, calculated based on the data from CATNAP in HIV-1 DATABASE. The “Overall average” refers to the percentage of antibodies in neutralizing each of the resistant strains separated according to CD4bs and non-CD4bs types. The other numbers represent the estimated means of IC_50_ and IC_80_ derived from at least three independent experiments. Values < 0.1 μg/ml are shown in red; 0.1–1 μg/ml in orange; 1–10 μg/ml in yellow; 10–50 μg/ml in green. Values > 50 μg/ml are shown in white. IDU: Intravenous drug use. R5: CCR5. X4: CXCR4. NA: not available. NC: negative control.

### Signature substitutions associated with broadly resistant HIV-1

**Fig 1:**
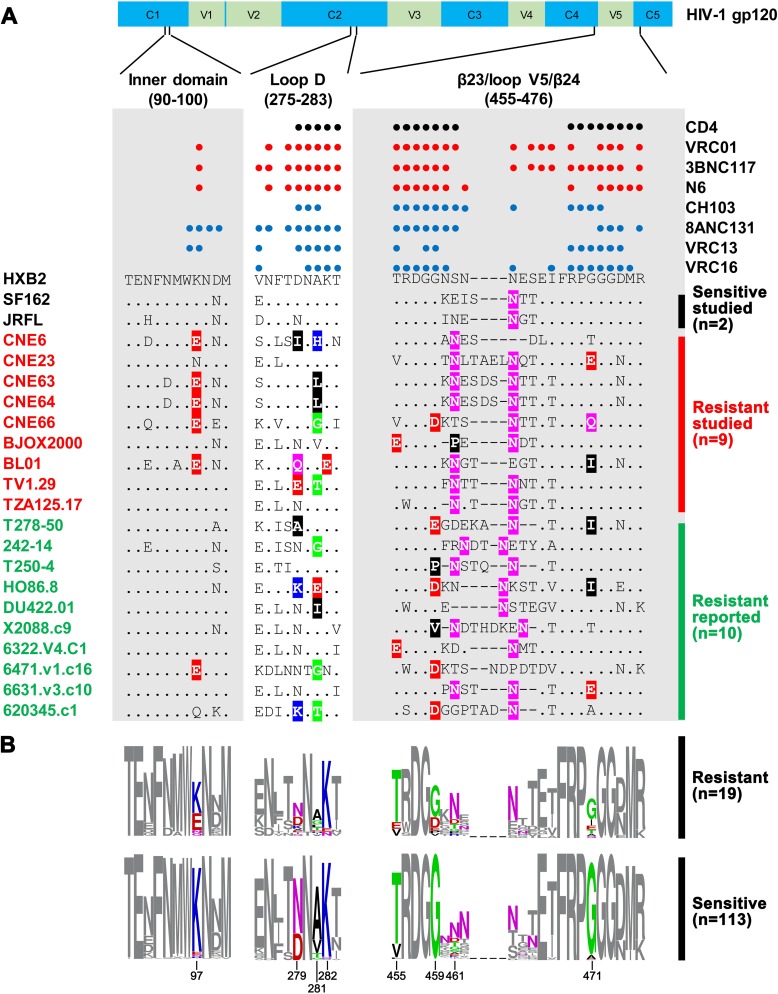
Signature substitutions distinguishing between the broadly resistant and sensitive HIV-1 strains in the contact regions of CD4bs bnAbs. **(A)**Alignment of the broadly resistant strains studied here (red, n = 9) and reported elsewhere (green, n = 10) against the standard sensitive strains HXB2, SF162 and JRFL.The locations of the contact regions such as the inner domain, Loop D and β23/loop V5/β24 along the HIV-1 gp120 and the actual contact residues are indicated above the aligned sequences. Each colored dot represents a single contact residue, with red for bnAbs of the VRC01 class (VRC01, 3BNC117 and N6) and blue for non-VRC01 classes (CH103, 8ANC131, VRC13 and VRC16). Residue differences from the HIV-1 HXB2 sequence are indicated by colors representing their different biochemical properties: green for polar, blue for basic, red for acidic, black for hydrophobic and purple for N or Q residues. Dots represent identical residues and dashes represent gaps introduced to preserve the alignment. **(B)** Identification of signature substitutions by quantitative comparisons between the broadly resistant strains and the sensitive ones using the program WebLogo. A total of 113 sensitive HIV-1 strains that can be neutralized by VRC01, 3BNC117, N6 and VRC13 were identified from reported research. The signature substitutions disproportionally represented among the resistant and sensitive strains are highlighted with their indicated locations. The colored scheme for the signature substitutions is the same as in (A). All residue numbers are based on the HIV-1 HXB2 sequence.

To identify residues potentially conferring broad resistance, we conducted comparative sequence analysis of resistant and sensitive strains available through the HIV Sequence Database (https://www.hiv.lanl.gov/) and published literature [17, 18, 27, 28].Here,broad resistance is defined as a strain resistant to at least 10 of the 16 CD4bs bnAbs tested with an IC_50_ of more than 50 μg/ml. Sensitive strains are those susceptible to at least VRC01, 3BNC117, N6 and VRC13 with an IC_50_ of less than 50 μg/ml.Based on these criteria, we identified a total of 19 broadly resistant strains and referred them as “Resistant panel to CD4bs bnAbs”,including the nine selected here for more thorough analysis (CNE6, CNE23, CNE63, CNE64, CNE66, BJOX2000, BL01, TV1.29 and TZA125.17). On the other hand, we found113 sensitive strains with diverse genetic and geographic backgrounds[17, 18, 27, 28].Sequence alignment of these 19 sequences against the standard sensitive strains HXB2, SF162 and JRFL revealed substantial differences of the CD4bs between the resistant and sensitive strains(Fig 1A).Most notable were changes in the inner domain, Loop D and β23/loop V5/β24 regions, which are critical for recognition by CD4bs bnAbs[17, 18, 29–31]. Resistant strains also had alterations in potential N-glycosylation sites, particularly in the β23/loop V5/β24 region (Fig 1A).We use the program WebLogo[51] to quantify altered residues in the epitope sequence that were disproportionally represented among the 19 resistant and 113 sensitive strains identified in the database (Fig 1B). These residues were primarily located in the inner domain at position 97, in Loop D at positions 279, 281 and 282, and in the β23/loop V5/β24 region at 455, 459, 461 and 471.For instance,at position 97, there was a significant enrichment of glutamic acid (E) among the resistant strains (31.6%) compared to the sensitive ones (3.5%).At position 281, alanine (A) was most prevalent among the sensitive strains (71.7%) but was reduced to 42.1% and largely replaced by glycine (G) (15.8%), leucine (L) (10.5%),or threonine (T)(10.5%) among the resistant strains.Similarly, the proportion of glycine (G) at positions 459 and 471 among the sensitive strains was severely reduced in the resistant ones, from 100% to 63.2% and 94.7% to 52.6% respectively.In particular, the potential N-glycosylation site at position 461, which was previously shown to conferresistance to VRC01 class antibodies [40, 52], increased its proportion from 23.0% among the sensitive strains to 42.1% among the resistant ones. These results suggest that multiple residues may be linked and act in unison to confer resistance, although the role for each residue is likely variable depending on the antibodies and actual strains they recognize.As many of these residues are located within antibody contact regions, the observed resistance likely results from reduced antibody binding, either due to the mutated residues themselves or steric hindrance imposed by the mutated residues.

### Signature substitutions conferring broad resistance

**Fig 2:**
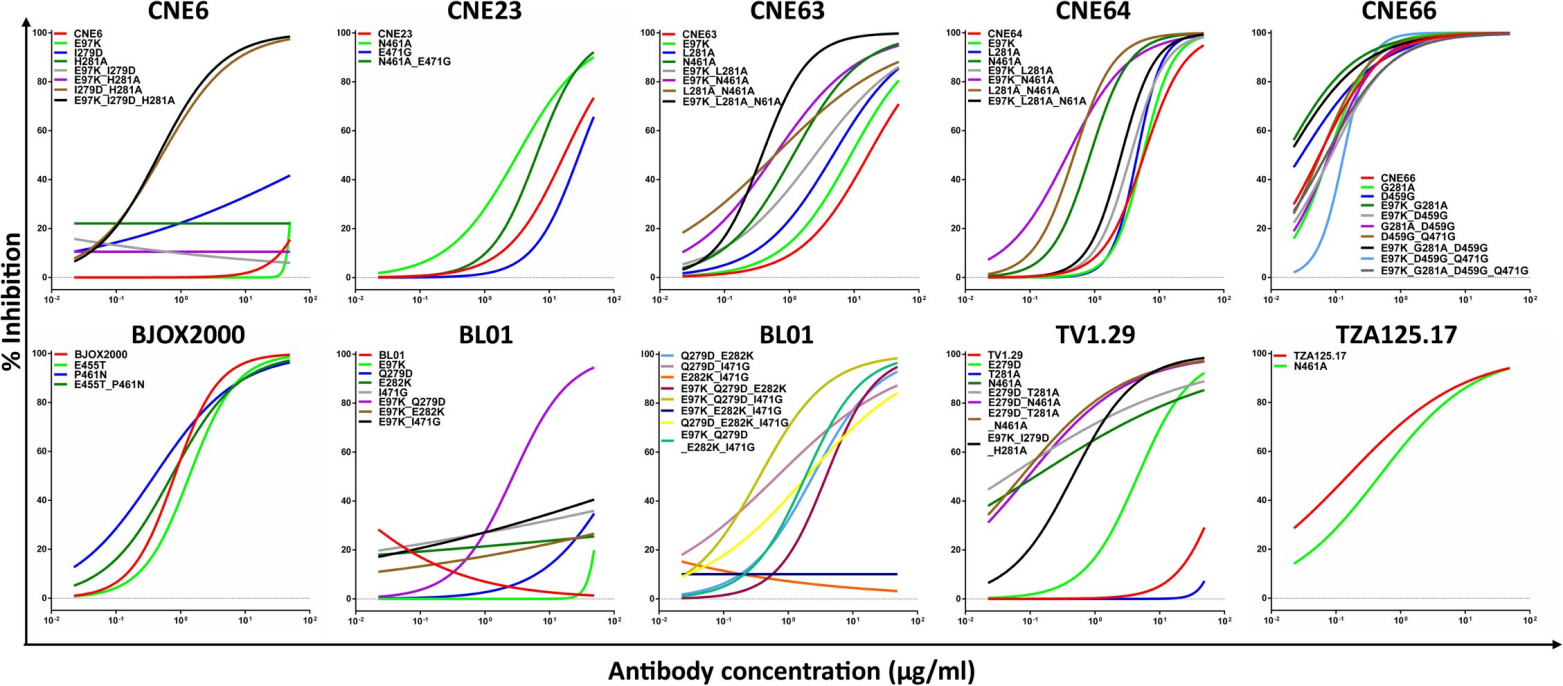
Neutralizing activity of N6 against wild-type and mutant HIV-1. Neutralization curves of N6 against nine wild-type and 58 mutant HIV-1 pseudoviruses. Data from one of three independent experiments is presented.

To study the role of the abovementioned residues in conferring resistance, we used site-directed mutagenesis to generate a total of 58 mutants of CNE6, CNE23, CNE63, CNE64, CNE66, BJOX2000, BL01, TV1.29 and TZA125.17 according to the consensus sensitive sequence highlighted in Fig 1B. The mutant envelopes included single, double, triple and quadruple substitutions,along with the various possible combinations thereof.Pseudoviruses bearing the mutant envelopes were then tested for sensitivity to representative antibodies from the VRC01-(VRC01, 3BNC117 and N6) and non-VRC01 classes (CH103, 8ANC131, VRC13 and VRC16).As predicted by sequence analysis, mutating the signature residues to the sensitive sequence restored sensitivity to the antibodies.However, the impact of each mutation varied significantly (Fig 2 and Table 2, under ‘Neutralizing activity’).Except in two instances where single residues fully restored sensitivity (CNE64_L281A and BJOX2000_E455T), the rest required multiple substitutions in one or more contact regions.For example, CNE6 required triple (E97K, I279D and H281A), CNE23 double (N461A and E471G), CNE63 double (L281A and N461A) and triple (E97K, L281A, and N461A), CNE64 double (E97K and L281A) and triple (E97K, L281A and N461A), CNE66 quadruple (E97K, G281A, D459G and Q471G), BJOX2000 double (E455T and P461N), BL01 triple (Q279D, E282K and I471G) and quadruple (E97K, Q279D, E282K and I471G), and TV1.19 double (E279D and N461A) and triple (E279D, T281A and N461A) substitutions to restore sensitivity to VRC01, 3BNC117 and N6,measured at an IC_50_ of less than 50 μg/ml (Table 2, left column under ‘Neutralizing activity’). Furthermore, different sets of triple substitutions were required to restore the sensitivity of CNE6 (E97K, I279D and H281A), CNE63 (E97K, L281A, and N461A), CNE64 (E97K, L281A and N461A), BL01 (Q279D, E282K and I471G), and TV1.29 (E279D, T281A and N461A)(Table 2, left column under ‘Neutralizing activity’).Furthermore, the impact of the same substitutions differed across different strains. For example, while double mutations at positions 97 and 281 failed to restore the sensitivity of CNE6 (N97K and H281A) to VRC01 and 3BNC117, they did so for CNE63 (N97K and L281A) and CNE64 (N97K and L281A), as measured by an IC_50_ of less than 50 μg/ml (Table 2, left column under ‘Neutralizing activity’). Furthermore, the double mutations (L281A and N461A) also had different effects on CNE63 and CNE64 in spite of their shared origin. In fact, the former was fully converted while the latter remained resistant to 3BNC117 (Table 2, left column under ‘Neutralizing activity’).These results support the hypothesis that multiple mutations acted in concert to confer resistance,although the specific residues required and their contributions varied from strain to strain.It needs to be noted that a single N461A substitution was sufficient to improve the sensitivity or revert resistance for some of the clones carrying the mutation (CNE23, CNE63,CNE64, TV1.29 and TZA125.17). This was particularly true for resistance to N6, but less so to VRC01, and not at all to 3BNC117 (Table 2, left column under ‘Neutralizing activity’).The role of N-glycosylation at 461 therefore differs in resistance against different antibodies.Steric hindrance from this particular glycosylation may hinder N6 from accessing its cognate epitope, but less so for VRC01 and was virtually negligible for 3BNC117.

**Table 2:** Restoration of the sensitive phenotype in broadly resistant HIV-1 strains through site-directed mutagenesis.

	Neutralizing activity																		Binding activity				Receptor usage	
	VRC01 class						non-VRC01 classes								CD4-related				VRC01 class			non-VRC01 classes	Cf2Th	Cf2Th
Virus strains and mutants	VRC01		3BNC117		N6		CH103		8ANC131		VRC13		VRC16		ibalizumab		CD4-Ig		VRC01	3BNC117	N6	VRC13	CD4.CCR5	CCR5
	IC_50_	IC_80_	IC_50_	IC_80_	IC_50_	IC_80_	IC_50_	IC_80_	IC_50_	IC_80_	IC_50_	IC_80_	IC_50_	IC_80_	IC_50_	IC_80_	IC_50_	IC_80_	GMFI			GMFI	RLU	RLU
CNE6	>50	>50	>50	>50	>50	>50	>50	>50	>50	>50	1.19	>50	>50	>50	0.19	0.99	5.41	14.36	10.41±2.96	11.66±2.74	11.29±2.62	16.43±1.72	2215341	27
CNE6_E97K	>50	>50	>50	>50	>50	>50	>50	>50	>50	>50	5.26	>50	>50	>50	0.23	2.10	3.29	10.14	10.29±2.99	12.26±3.41	10.73±2.28	15.73±1.37	5684407	36
CNE6_I279D	1.10	15.57	28.15	>50	>50	>50	>50	>50	>50	>50	0.04	0.67	>50	>50	0.05	0.14	40.90	>50	13.53±3.25	12.78±3.23	11.76±2.94	17.43±1.78	1523709	27
CNE6_H281A	40.83	>50	>50	>50	>50	>50	>50	>50	>50	>50	1.38	16.54	>50	>50	0.02	0.06	>50	>50	10.45±3.01	10.87±2.71	11.53±2.78	16.50±2.66	431281	13
CNE6_E97K_I279D	1.98	4.82	5.99	23.50	>50	>50	>50	>50	>50	>50	0.33	1.83	>50	>50	0.09	0.23	29.91	>50	15.10±3.54	15.23±3.71	11.66±2.55	17.50±2.83	2943819	10
CNE6_E97K_H281A	>50	>50	>50	>50	>50	>50	>50	>50	>50	>50	9.43	>50	>50	>50	0.04	0.07	>50	>50	11.00±3.46	11.35±3.15	12.66±3.75	15.83±1.88	113754	15
CNE6_I279D_H281A	28.72	>50	9.50	>50	0.51	2.91	>50	>50	>50	>50	0.11	1.13	>50	>50	0.58	2.30	3.04	10.11	14.01±3.86	15.17±3.31	18.75±2.45	16.93±2.55	3942741	32
CNE6_E97K_I279D_H281A	12.18	26.50	1.25	4.20	0.45	2.16	>50	>50	>50	>50	0.67	2.20	>50	>50	3.12	>50	1.56	4.23	14.94±4.51	17.47±4.13	19.90±4.10	17.73±3.10	3237033	32
CNE23	41.87	>50	>50	>50	16.94	>50	>50	>50	>50	>50	>50	>50	>50	>50	0.02	0.06	>50	>50	10.67±2.00	12.67±0.82	12.70±0.50	14.45±2.05	1199356	24
CNE23_N461A	8.34	24.83	>50	>50	3.19	14.78	>50	>50	>50	>50	2.55	>50	>50	>50	0.04	>50	>50	>50	12.13±0.92	13.60±1.57	16.34±2.24	14.85±0.15	1662466	51
CNE23_E471G	>50	>50	>50	>50	28.74	>50	>50	>50	>50	>50	4.02	>50	>50	>50	0.03	0.08	11.65	34.87	10.86±1.32	13.37±0.69	12.61±0.39	15.85±1.55	2482645	59
CNE23_N461A_E471G	8.27	33.34	13.76	41.92	6.36	20.02	>50	>50	>50	>50	2.25	31.52	>50	>50	0.10	>50	6.05	43.48	12.60±1.55	14.87±1.35	16.26±1.16	16.35±1.05	2669833	39
CNE63	>50	>50	>50	>50	16.50	>50	>50	>50	>50	>50	6.74	44.19	>50	>50	0.01	0.03	>50	>50	9.18±1.17	9.98±0.84	10.35±0.80	15.73±1.58	87966	32
CNE63_E97K	>50	>50	>50	>50	8.85	46.91	>50	>50	>50	>50	5.90	35.14	>50	>50	0.01	0.04	13.15	>50	9.44±1.27	10.31±1.27	10.60±0.78	15.60±1.36	85643	23
CNE63_L281A	22.98	>50	21.62	>50	4.73	29.35	>50	>50	>50	>50	1.38	13.78	>50	>50	0.01	0.04	21.28	>50	9.84±1.11	10.89±1.09	10.62±0.76	15.67±1.93	49487	19
CNE63_N461A	24.27	>50	>50	>50	1.15	6.24	>50	>50	>50	>50	4.09	18.65	>50	>50	0.02	0.17	9.97	>50	10.16±1.11	10.46±0.96	10.99±1.28	14.47±1.52	123704	15
CNE63_E97K_L281A	11.70	>50	13.85	44.62	2.49	24.11	>50	>50	>50	>50	0.81	7.63	>50	>50	0.01	0.03	10.24	>50	9.69±1.31	10.62±1.23	10.83±0.52	14.03±1.01	271723	18
CNE63_E97K_N461A	0.03	17.40	>50	>50	0.86	3.72	>50	>50	>50	>50	1.96	19.37	>50	>50	0.02	0.90	4.81	>50	10.19±0.95	10.32±0.82	11.01±1.23	14.30±1.82	200466	97
CNE63_L281A_N461A	3.03	15.79	6.95	31.89	2.01	6.38	>50	>50	>50	>50	0.38	4.18	>50	>50	0.02	0.35	8.50	>50	10.42±0.96	11.30±0.83	10.54±1.30	14.90±1.71	49046	24
CNE63_E97K_L281A_N461A	2.21	5.62	0.30	3.52	0.38	1.19	>50	>50	33.48	>50	0.12	1.33	1.35	6.68	0.11	26.63	1.10	3.22	12.03±0.63	12.93±1.52	13.63±1.43	15.73±1.01	293879	21
CNE64	>50	>50	>50	>50	5.82	16.49	>50	>50	>50	>50	0.02	0.46	>50	>50	0.08	0.47	3.11	17.59	9.54±1.31	9.99±1.17	11.07±0.05	13.83±1.16	24957	64
CNE64_E97K	>50	>50	>50	>50	5.62	11.70	>50	>50	>50	>50	0.15	3.59	>50	>50	0.17	0.44	3.26	8.00	9.85±1.32	10.46±1.36	13.03±1.74	13.97±1.67	76618	45
CNE64_L281A	14.52	36.67	6.23	31.10	4.56	8.47	>50	>50	>50	>50	0.08	1.07	45.95	>50	0.04	0.10	12.50	30.24	10.12±1.37	11.10±1.31	12.43±1.18	14.63±1.51	227521	64
CNE64_N461A	>50	>50	>50	>50	0.85	2.24	>50	>50	>50	>50	0.10	1.32	>50	>50	3.47	>50	3.22	7.22	11.15±1.78	10.61±1.71	14.57±1.79	15.13±2.70	66595	47
CNE64_E97K_L281A	3.47	33.68	2.20	14.11	3.63	8.60	>50	>50	>50	>50	0.20	3.60	37.16	>50	0.06	0.16	1.54	6.36	9.15±1.15	10.19±1.41	12.33±0.47	14.35±3.25	447707	56
CNE64_E97K_N461A	10.97	31.34	>50	>50	0.38	1.75	>50	>50	>50	>50	0.43	4.87	>50	>50	0.20	28.56	0.56	1.55	11.10±1.45	10.65±1.65	15.20±2.83	14.27±1.75	122324	34
CNE64_L281A_N461A	1.76	6.60	>50	>50	0.48	1.30	>50	>50	>50	>50	0.15	1.88	1.28	5.67	0.48	44.64	5.68	9.54	11.30±0.93	10.79±1.19	15.07±3.63	13.97±1.36	352783	36
CNE64_E97K_L281A_N461A	3.33	9.85	2.38	13.38	2.59	5.88	>50	>50	>50	>50	2.10	39.06	>50	>50	0.13	2.02	>50	>50	11.37±0.73	12.38±2.15	17.60±3.11	13.47±1.05	227625	27
CNE66	>50	>50	>50	>50	0.06	0.25	>50	>50	>50	>50	>50	>50	>50	>50	0.05	0.38	22.75	>50	8.22±0.96	9.49±0.05	13.55±0.05	10.75±0.55	9162	16
CNE66_G281A	>50	>50	>50	>50	0.08	0.22	>50	>50	>50	>50	>50	>50	>50	>50	0.14	1.08	2.08	>50	7.95±0.64	9.22±0.40	13.75±0.75	11.05±0.85	522912	82
CNE66_D459G	16.90	>50	>50	>50	0.03	0.22	2.12	16.61	>50	>50	>50	>50	>50	>50	0.04	0.85	1.74	>50	8.52±1.12	9.33±0.11	12.30±0.20	10.80±0.50	88661	24
CNE66_E97K_G281A	9.32	>50	>50	>50	0.02	0.09	>50	>50	>50	>50	>50	>50	>50	>50	0.05	0.48	1.16	42.74	8.03±0.60	9.28±0.45	13.60±1.30	10.68±0.93	44540	18
CNE66_E97K_D459G	>50	>50	>50	>50	0.09	0.38	>50	>50	>50	>50	>50	>50	>50	>50	0.12	0.36	2.38	18.06	8.37±0.84	9.50±0.01	11.50±0.70	10.32±0.69	59181	16
CNE66_G281A_D459G	2.89	7.22	>50	>50	0.08	0.27	5.39	36.82	>50	>50	42.85	>50	>50	>50	0.05	1.78	1.48	10.92	9.99±0.51	9.74±0.04	12.85±0.05	11.15±0.45	48345	18
CNE66_D459G_Q471G	>50	>50	>50	>50	0.07	0.18	9.25	>50	>50	>50	0.34	6.41	>50	>50	0.14	0.31	>50	>50	8.97±1.44	12.15±1.05	15.80±1.80	12.85±1.05	23815	21
CNE66_E97K_G281A_D459G	1.18	16.36	>50	>50	0.02	0.12	2.40	25.32	>50	>50	>50	>50	>50	>50	0.08	1.65	0.69	4.65	9.62±0.98	10.08±0.73	14.70±1.60	11.40±0.40	62833	18
CNE66_E97K_D459G_Q471G	2.80	41.18	>50	>50	0.13	0.25	>50	>50	>50	>50	6.36	29.98	>50	>50	0.02	0.10	5.22	>50	9.27±1.43	11.70±1.30	15.10±2.40	12.35±0.75	19326	22
CNE66_E97K_G281A_D459G_Q471G	0.58	4.17	0.23	3.97	0.07	0.36	5.97	20.48	1.54	13.15	5.97	19.87	>50	>50	0.07	0.19	3.32	36.55	10.77±0.84	13.15±1.75	15.25±1.75	13.10±1.30	6224	22
BJOX2000	>50	>50	>50	>50	0.81	2.38	>50	>50	18.13	>50	>50	>50	>50	>50	0.09	>50	31.84	>50	11.40±0.50	11.30±0.30	14.65±0.05	11.70±0.40	87876	36
BJOX2000_E455T	10.08	25.44	9.17	19.07	1.37	5.79	>50	>50	34.29	>50	48.12	>50	>50	>0	0.04	>50	13.90	>50	12.40±0.40	13.15±0.35	14.75±0.05	12.00±0.10	217493	39
BJOX2000_P461N	>50	>50	>50	>50	0.39	3.07	0.64	24.57	>50	>50	>50	>50	>50	>50	0.06	>50	>50	>50	11.75±0.05	11.65±0.05	15.55±0.75	11.60±0.60	419246	61
BJOX2000_E455T_P461N	2.29	11.90	1.80	10.81	0.73	3.81	19.49	>50	>50	>50	>50	>50	24.63	>50	0.03	>50	5.27	>50	10.75±0.45	11.70±0.50	13.40±0.60	12.60±0.60	2120789	5583
BL01	>50	>50	>50	>50	>50	>50	>50	>50	>50	>50	>50	>50	>50	>50	0.08	8.85	2.10	14.26	ND	ND	ND	ND	537355	26
BL01_E97K	>50	>50	>50	>50	>50	>50	>50	>50	>50	>50	>50	>50	>50	>50	0.15	19.70	2.25	8.29	ND	ND	ND	ND	555891	31
BL01_Q279D	42.98	>50	>50	>50	>50	>50	>50	>50	>50	>50	>50	>50	>50	>50	0.10	0.39	5.64	44.80	ND	ND	ND	ND	34957	31
BL01_E282K	>50	>50	>50	>50	>50	>50	>50	>50	>50	>50	>50	>50	>50	>50	0.14	>50	2.06	12.78	ND	ND	ND	ND	208063	51
BL01_I471G	>50	>50	>50	>50	>50	>50	>50	>50	>50	>50	>50	>50	>50	>50	0.05	0.34	12.11	39.53	ND	ND	ND	ND	421749	26
BL01_E97K_Q279D	11.39	>50	>50	>50	2.70	10.98	>50	>50	>50	>50	>50	>50	>50	>50	0.09	3.08	1.79	7.97	ND	ND	ND	ND	115835	22
BL01_E97K_E282K	>50	>50	>50	>50	>50	>50	>50	>50	>50	>50	>50	>50	>50	>50	0.04	>50	0.69	11.72	ND	ND	ND	ND	469409	78
BL01_E97K_I471G	>50	>50	>50	>50	>50	>50	>50	>50	>50	>50	>50	>50	>50	>50	0.07	2.09	1.86	14.94	ND	ND	ND	ND	199279	87
BL01_Q279D_E282K	40.68	>50	>50	>50	2.39	12.20	>50	>50	>50	>50	>50	>50	>50	>50	0.06	>50	2.30	9.14	ND	ND	ND	ND	470740	27
BL01_Q279D_I471G	31.02	>50	>50	>50	0.67	19.48	>50	>50	>50	>50	>50	>50	>50	>50	0.07	0.15	28.43	>50	ND	ND	ND	ND	136232	34
BL01_E282K_I471G	>50	>50	>50	>50	>50	>50	>50	>50	>50	>50	>50	>50	>50	>50	0.04	0.59	8.26	>50	ND	ND	ND	ND	354475	26
BL01_E97K_Q279D_E282K	19.86	>50	>50	>50	3.86	12.97	>50	>50	>50	>50	>50	>50	>50	>50	0.17	>50	0.95	4.38	ND	ND	ND	ND	413664	21
BL01_E97K_Q279D_I471G	25.49	>50	>50	>50	0.36	1.70	>50	>50	>50	>50	>50	>50	>50	>50	0.09	1.08	16.30	43.57	ND	ND	ND	ND	197560	51
BL01_E97K_E282K_I471G	>50	>50	>50	>50	>50	>50	>50	>50	>50	>50	>50	>50	>50	>50	0.17	>50	3.09	20.72	ND	ND	ND	ND	724553	44
BL01_Q279D_E282K_I471G	31.62	>50	0.52	9.83	1.94	28.51	>50	>50	>50	>50	>50	>50	>50	>50	0.13	16.92	6.06	28.75	ND	ND	ND	ND	253740	34
BL01_E97K_Q279D_E282K_I471G	23.33	>50	0.79	6.75	1.85	7.33	>50	>50	>50	>50	>50	>50	>50	>50	0.07	>50	2.71	14.22	ND	ND	ND	ND	737429	31
TV1.29	>50	>50	>50	>50	>50	>50	>50	>50	>50	>50	0.06	0.87	>50	>50	0.02	0.10	0.56	2.72	ND	ND	ND	ND	118590	44
TV1.29_E279D	>50	>50	18.80	45.08	5.50	15.50	>50	>50	>50	>50	5.26	>50	>50	>50	0.03	0.08	0.60	5.32	ND	ND	ND	ND	268497	70
TV1.29_T281A	>50	>50	>50	>50	>50	>50	>50	>50	>50	>50	0.03	0.09	>50	>50	0.01	0.09	6.97	19.27	ND	ND	ND	ND	12582	34
TV1.29_N461A	>50	>50	>50	>50	0.12	16.50	>50	>50	>50	>50	0.81	2.96	>50	>50	>50	>50	0.71	1.56	ND	ND	ND	ND	87462	34
TV1.29_E279D_T281A	>50	>50	20.20	>50	0.05	4.72	>50	>50	>50	>50	0.09	1.12	>50	>50	0.04	0.09	2.13	13.23	ND	ND	ND	ND	206570	36
TV1.29_E279D_N461A	0.74	10.29	0.90	6.83	0.09	1.12	>50	>50	>50	>50	0.87	4.17	>50	>50	0.16	>50	1.29	5.38	ND	ND	ND	ND	372688	27
TV1.29_E279D_T281A_N461A	1.37	5.29	2.75	24.18	0.07	0.91	>50	>50	>50	>50	0.98	3.93	>50	>50	37.38	>50	5.82	24.63	ND	ND	ND	ND	117690	31
TZA125.17	>50	>50	>50	>50	0.23	2.30	>50	>50	>50	>50	>50	>50	>50	>50	0.03	0.61	>50	>50	ND	ND	ND	ND	28008	34
TZA125.17_N461A	25.80	>50	>50	>50	0.47	5.51	>50	>50	>50	>50	>50	>50	>50	>50	0.73	3.46	17.16	>50	ND	ND	ND	ND	67566	34
**Meidian IC**_**50**_**/IC**_**80**_**(μg/ml)**	<0.1	0.1–1	1–10	10–50	>50				**n-fold of GMFI**		0.5–1.2	>1.2				**RLU**		<10^3^	10^3^−10^4^	10^4^−10^5^	10^5^−10^6^	10^6^−10^7^		

The numbers under “Neutralizing activity” represent the estimated mean values of IC_50_ and IC_80_ derived from at least three independent experiments. The color scheme is the same as in Table 1. The numbers under “Binding activity” represent the means and variation of estimated GMFI values from at least three independent experiments. Those that increased more than 1.2-fold relative to the original clones are indicated in blue. The numbers under ‘Receptor usage’ represent the luciferase activity in relative light units (RLU). The color scheme represents various ranges of RLU indicated below. ND: not detected.

Among the non-VRC01 class antibodies tested, VRC13 stands out in neutralization of the wild type and mutant strains carrying the signature substitutions, except for BL01 and TZA125.17 (Table 2, left column under ‘Neutralizing activity’). Other antibodies in this group, such as CH103, 8ANC131 and VRC16, remained unable to neutralize most mutated clones, except for a few examples among the CNE63, CNE64, CNE66 and BJOX2000 mutants (Table 2, the left column under ‘Neutralizing activity’). The signature mutations therefore contributed little to this class of CD4bs antibodies. Other factors such as mutations beyond those tested or the angle of antibody approaching the epitope likely play more significant roles. Lastly, some mutant strains showed a drastic change in sensitivity to the CD4-specific antibodies ibalizumab and CD4-Ig, suggesting that the signature mutations alter the interaction between the envelope and CD4 receptor. For example, the N461A mutation in CNE23, CNE64 and TV1.29 greatly reduced or completely abolished neutralizing sensitivity to ibalizumab. This was consistent with a previous report that a glycan is crucial for ibalizumab recognition [53, 54]. Significant reduction in ibalizumab neutralization activity also occurred in many of the mutant clones, which are highlighted in Table 2 (left column under ‘Neutralizing activity’).

Signature substitutions appear to alter sensitivity to CD4-Ig. Complete conversion from CD4-Ig sensitive to resistant was observed in mutants CNE6_H281A, CNE6_E97K_H281A, CNE64_E97K_L281A_N461A, CEN66_D459G_Q471G and BJOX2000_P461N. The opposite conversion was also found for many mutants derived from CNE23, CNE63 and TZA125.17 highlighted in yellow and green (Table 2, left column under ‘Neutralizing activity’). However, all of the wild-type and mutant strains maintained dependence on the CD4 receptor and CCR5 co-receptor for entry, except for BJOX2000_E455T_P461N. In this case, the CCR5 co-receptor alone was sufficient, albeit with significantly reduced entry efficiency (2,120,789 vs. 5,583) (Table 2, right column under ‘Receptor usage’). Consistent with previous reports that fitness cost is associated with escape from CD4bs antibodies [30, 55], we found a compromised entry potential in 3 broadly resistant strains (CNE63, CNE64, and BJOX2000). However, the remaining 6 (CNE6, CNE23, CNE66, BL01, TV1.29, and TZA125.17) showed no appreciable differences compared to their maximally converted sensitive counterparts (Table 2, under ‘Receptor usage’). Taken together, these results highlight that the signature substitutions within antibody contact sites are critical determinants of the viral strains’ resistance phenotype. This was particularly true for VRC01 class and less so for non-VRC01 class antibodies.Some signature substitutions also appear to alter the interaction between the envelope and CD4 molecule and affect viral entry efficiency, highlighting the structural plasticity and functional diversity of CD4bs that facilitates binding to the cellular receptor and entry into the target cells.

### Reverting signature substitutions improved antibody binding to envelope trimers on the cell surface

To study the potential mechanisms by which the signature substitutions restore antibody sensitivity, we first compared their effect on antibody binding to envelope trimers. HEK293T cells were transfected with a total of 42original and mutated envelope clones from CEN6, CNE23, CNE63, CNE64, CNE66 and BJOX2000 to express HIV-1 trimers on the cell surface and then stained separately with VRC01, 3BNC117, N6 and VRC13 at 10μg/ml, together with appropriate background controls such as mock-transfected HEK293T cells and envelope-transfected cells stained with the secondary conjugated antibody. The gating strategy was properly adjusted according to the controls before the geometric mean fluorescence intensity (GMFI) and variation were estimated based on the 3 repeat experiments for the tested antibodies (Table 2, under ‘Binding activity’ and S1 Fig). Consistent with neutralization studies, improved antibody binding predominantly occurred in clones with multiple substitutions in one or more contact regions. For example, N6 was found to have enhanced binding activity towards the double (I279D_H281A) and triple (E97K_I279D_H281A) mutants of CNE6, the double (N461_E471G) mutant of CNE23, the triple (E97K_L281A_N461A) mutant of CNE63, and the double (E97K_N461A, L281A_N461K) and triple (E97K_L281A_N461A) mutants of CNE64 (Table 2, under ‘Binding activity’). In particular, triple mutations in CNE6(E97K_I279D_H281A) and CNE63 (E97K_L281A_N461A) also led to enhanced binding to VRC01 and 3BNC117 (Table 2, under ‘Binding activity’). Similarly, the triple mutations (E97K_L281A_N461A) in CNE64 improved binding to 3BNC117 while the quadruple mutations (E97K, G281A, D459G and Q471G) in CNE66 improved that to VRC01 and 3BNC117 (Table 2, under ‘Binding activity’).

Single mutations in a few trimers also improved binding with the tested antibodies. Of these, CNE6_I279D, CNE23_N461A, and CNE64_N461A demonstrated enhanced binding with either VRC01 or N6 (Table 2, under ‘Binding activity’). This suggests that the glycan at position 461 may contribute, at least partially,to the resistance of some clones. However, for some of the single mutations within the predicted contact sites that restored neutralization activity, no measurable changes in binding were identified. This is perhaps not surprising, as these mutations may not contribute substantial binding energy to the overall interaction. The role of individual residues is also expected to vary depending on the envelope context. Of note, while the binding to VRC01, 3BNC117 and N6 was enhanced for a large proportion of the mutant trimers, the impact on VRC13 activity was relatively smaller. The highest improvement was found for CNE66 trimer with quadruple mutations (E97K, G281A, D459G and Q471G) (Table 2, under ‘Binding activity’).

**Fig 3:**
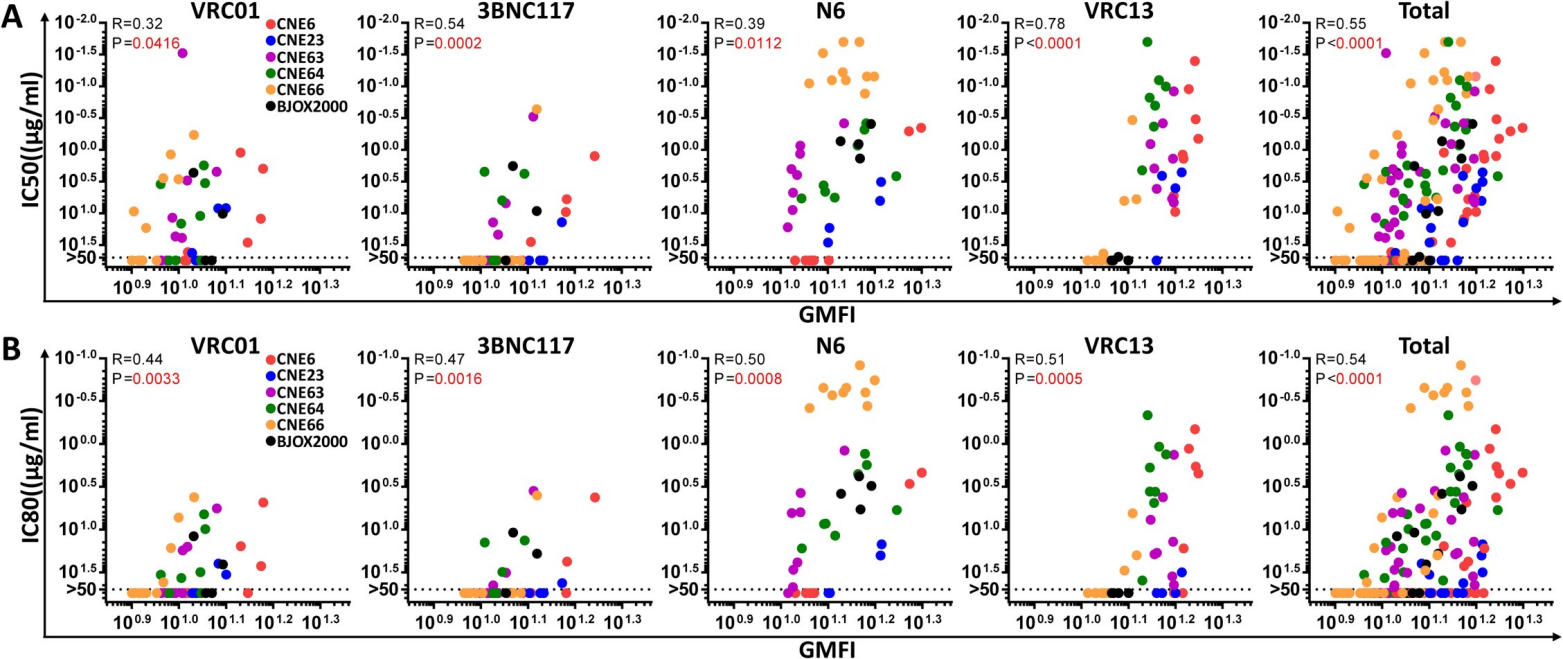
Correlations between the neutralizing activity of bnAbs(IC_50_ in (A) and IC_80_ in (B)) and binding to the Env trimers expressed on the cell surface. Correlations for each and all antibodies together (far right) were analyzed by determining the Spearman correlation coefficient (R), with statistical significance (P) indicated in red. Different resistant clones and their derivatives are indicated by different colors.

Next, we conducted correlation analysis between cell surface binding and neutralizing activity (Fig 3). When all data from all tested antibodies were combined, we found a significant and direct correlation between cell surface binding and neutralizing activity for both IC_50_ and IC_80_ (P < 0.0001; Spearman’s R = 0.55 and 0.54respectively) (Fig 3). For each individual antibody, significant correlation between cell surface binding and neutralizing activity was also identified.Taken together, these findings support the idea that the majority of mutations under study restore neutralization sensitivity largely by enhancing the binding of the antibodies to their respective target epitopes. Steric disruption likely plays a predominant role for cases where removal of the glycan at position 461 improved binding and neutralization.

### Signature substitutions transformed HIV-1 from sensitive to resistant phenotype

**Fig 4:**
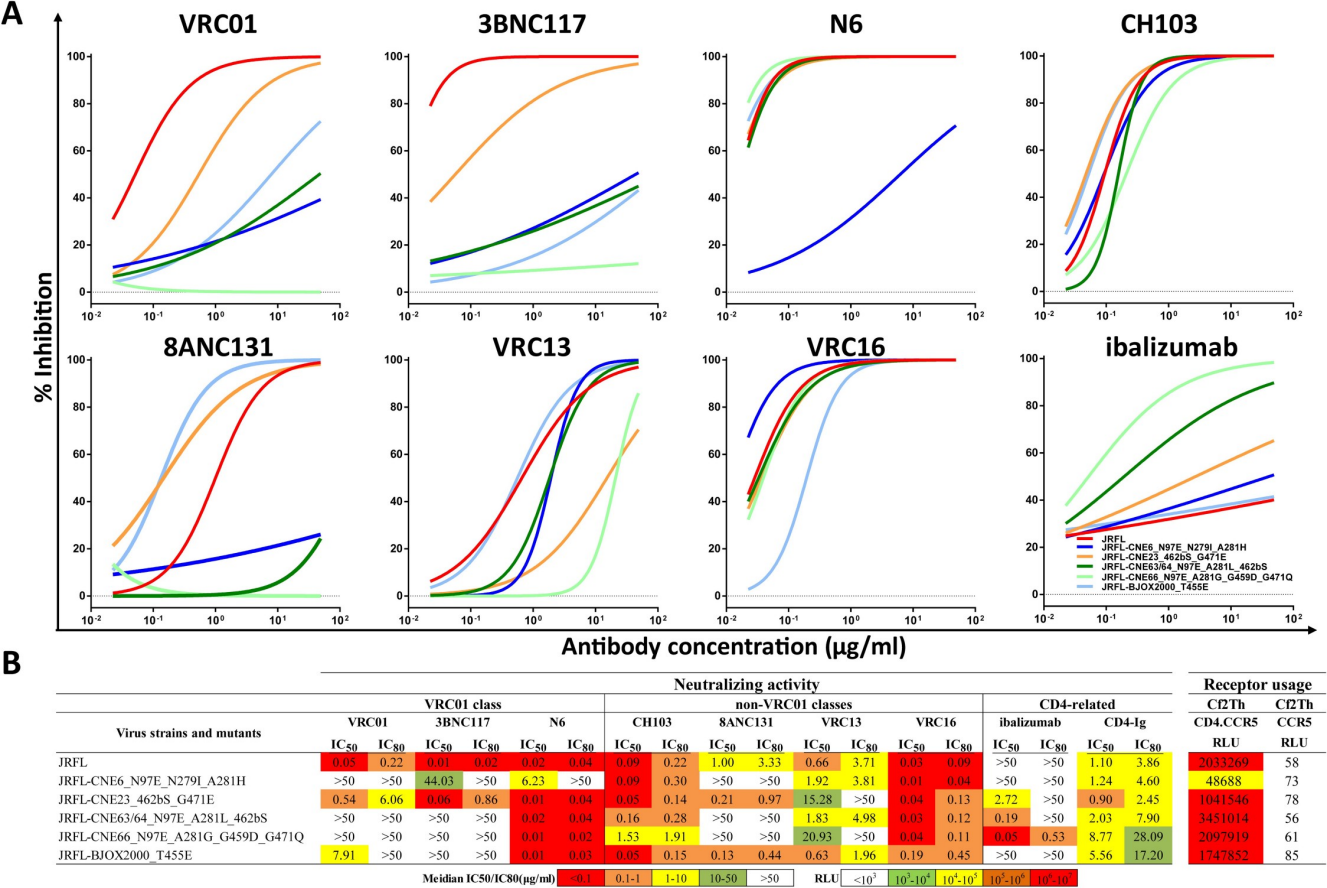
Introduction of signature substitutions transformed the sensitive HIV-1 JRFL into the resistant phenotype. **(A)** Neutralization curves of VRC01 class, non-VRC01 class antibodies and ibalizumab against JRFL mutants containing a combination of signature substitutions from CNE6, CNE23, CNE63/64, CNE66 and BJOX2000. Data from one of three independent experiments is presented. **(B)** IC_50_ and IC_80_ values for each of the tested antibodies against wild-type and mutant HIV-1 JRFL. Receptor usage based on Cf2Th cell lines is also indicated. All data is presented as the means of at least three independent experiments. The color scheme used is identical to that in Tables 1 and 2.

To further analyze how the substitutions under study act to restore antibody sensitivity, we introduced several residue combinations into envelope clone JRFL, an HIV-1 strain sensitive to the tested CD4bs antibodies. We hypothesized that incorporating resistance-conferring substitutions into the envelope of a sensitive strain of HIV-1 can convert it to a resistant phenotype. To test this, combinations of signature substitutions matching those in CNE6, CNE23, CNE63, CNE64, CNE66 and BJOX2000 were introduced into the JRFL envelope. For strains altered to match CNE23, CNE63 and CNE64, an extra serine residue (S) was added to the β23/loop V5/β24 region to convert asparagine (N) at position 461 into a potential glycosylation site.As shown in Fig 4, pseudoviruses bearing these mutated JRFL envelopes had significantly improved resistance to the bnAbs from both the VRC01 and non-VRC01 classes. The substitutions appeared to be more effective in conferring resistance to VRC01, 3BNC117, 8ANC131, and VRC13.Only JRFL-CNE6_N97E_N279I_A281H and JRFL-CNE66_N97E_A281G_G459D_G471Q demonstrated significantly enhanced resistance to N6 (312-fold) and CH103 (17-fold) respectively, as measured by IC_50_.As previously discussed, this was not surprising since we expect that envelope clones require different sets of substitutions to confer resistance and the relative role of each substitution may vary in different envelope contexts. Given that JFRL is a model virus for sensitivity to the tested antibodies, additional substitutions may be needed to generate broader levels of resistance. Interestingly, clones that are fully resistant to VRC01, 3BNC117 and 8ANC131 derived from CNE63, CNE64 and CNE66 inadvertently became highly sensitive to ibalizumab.The addition of an additional glycan at position 461 in JRFL-CNE63/64_N97E_A281L_462bS may provide the structural support required for ibalizumab recognition. Similar reasoning could also explain the improved sensitivity of JRFL-CNE23_462bS_G471E to ibalizumab.However, the sensitivity in JRFL-CNE66_N97E_A281G_G459D_G471Q remains uncertain. Lastly, entry of practically all the mutant JRFL variants into the target cell remained dependent on the CD4 receptor and CCR5 co-receptor. The only exception was JRFL-CNE6_N97E_N279I_A281H, whose entry efficiency was reduced by approximately 42-fold relative to the original JRFL (48,688 vs. 2,033,269) (Fig 4B, under ‘Receptor usage’).

### Structural basis for resistance conferred by signature substitutions

**Fig 5:**
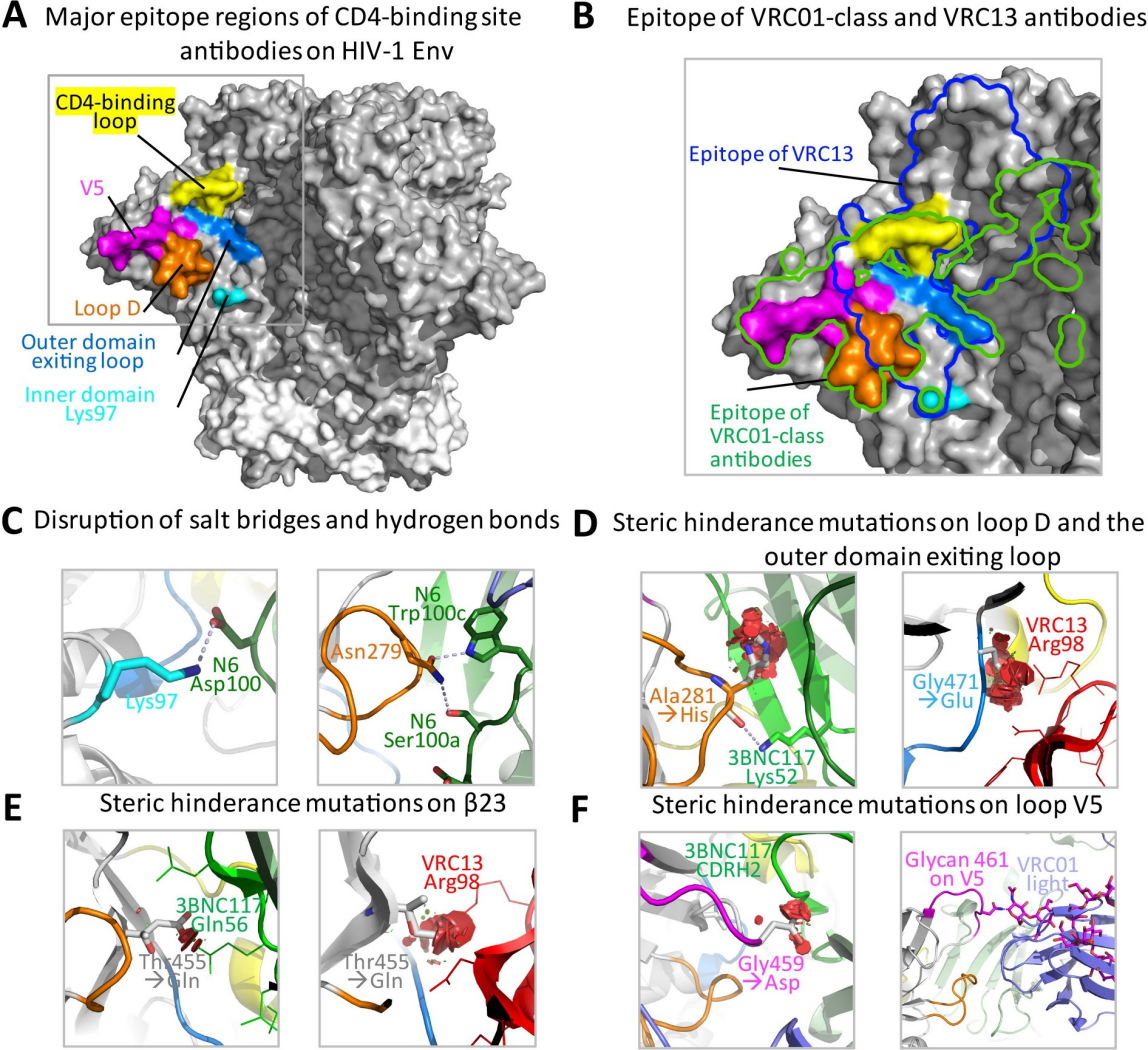
Structural basis of the signature resistance mutations. **(A)** Major epitope regions of CD4-binding site antibodies on the HIV-1 Env in its pre-fusion closed conformation. The inner domain contact, Loop D, CD4-binding loop, Loop V5 and the outer domain exiting loop are colored cyan, orange, yellow, magenta and marine blue respectively. **(B)** Overlay of the boundaries of epitopes for VH1-2-derived VRC01-class antibodies (green line) and VRC13 (blue line) onto the different structural regions. **(C)** Resistance conferred by mutations that disrupt salt bridges and hydrogen bonds between gp120 and CD4bs antibodies. HIV-1 gp120 residues were colored and labeled according to their structural regions and the antibody residues were labeled by source and location, such as Asp100 of antibody N6. **(D)** Resistance conferred by mutations providing steric hinderance on loop D and the outer domain exiting loop. Potential clashes between resistance mutations and the antibodies were marked as red discs. The heavy chains of VRC01 class antibodies were colored in shades of green and CDR H3 of VRC13 was colored red. Light chains were colored slate. **(E)** Resistance conferred by mutations providing steric hinderance on β23. **(F)** Resistance conferred by mutations providing steric hinderance through bulkier residues or a glycan on loop V5.

The supersite on HIV-1 gp120 targeted by CD4bs antibodies is composed of multiple areas, including loop D, the CD4-binding loop, loop V5, the outer domain exiting loop and small patches on the inner domain (Fig 5A). The signature mutations that confer resistance to CD4-binding site antibodies arose in these regions (Fig 1).To explain the structural basis for these substitutions, we analyzed the resistance-related mutations using the pre-fusion closed HIV-1 Env structure with CD4bs antibodies bound (PDB IDs: 5FYJ for VRC01 and 5V8M for 3BNC117) or docked from their Fab-gp120 complexes by aligning the gp120 outer domains (PDB IDs: 5TE6 for gp120-bound N6, 4YDJ for gp120-bound VRC13, 4RWY for gp120-bound 8ANC131).A comparison of the epitope of VRC01 class antibodies and that of VRC13 indicated significant overlap centered at the CD4-binding loop and outer domain exiting loop (Fig 5B). Analysis of contact residues indicated that inner domain residue Lys97 (92.3% conserved)made salt bridges with CDR H3 residues Asp99 in VRC01 and Asp100 in N6 (Fig 5C). It is of note that a Lys to Glu mutation occurred at this position in the resistant strains CNE6, CNE63, CNE64, CNE66 and BL01 (Fig 1A). The loop D residue 279 is highly conserved as either an Asp or Asn (97.1% conservation according to the LANL HIV-1 database) that forms a hydrogen bond with the conserved Trp in CDR H3 of VRC01 class antibodies (Trp100b in VRC01, Trp100 in 3BNC117 and Trp100c in N6). Mutations of other residues abolished this critical hydrogen bond (Fig 5C) and probably contributed to the resistance phenotype of strains CNE6, BL01 and TV1.29. Conversely, reverting to Asp279 in CNE6 and TV1.29 conferred sensitivity to VRC01 class antibodies (Table 2). Disruption of key salt bridges and hydrogen bonds with CD4bs antibodies is apparently one of the factors that contributed to the resistance phenotype.

On the other hand, a hydrogen bond with a conserved Lys52 in CDR H2 of VRC01 class antibodies was made by loop D residue 281 through its main chain O atom, which seemed to be independent of side-chain mutations. However, mutation to amino acids with bulkier side chains at this location, such as His, Leu and Glu, occurred in strains CNE6, CNE63, CNE64 and HO086.8, which caused steric hindrance for VRC01 class antibodies and hence conferred resistance (Fig 5D). Structural data indicated that a residue with a short side chain, such as an Ala, is preferred in sensitive strains (77.8% Ala, 11.5% Thr and 6.6% Val). Mutations that provided steric hindrance to antibody binding also occurred at several other positions, such as β23 residue 455, loop V5 residue 459and outer domain exiting loop residue 471 (Fig 5D–5F). Reverting Glu455 in strain BJOX2000 to Thr (90.6% conserved) restored sensitivity to VRC01 (Table 2).A change of Gly459 (96.9% conserved) to other bulkier residues occurred in 7 of the 19 resistant strains (Fig 1). In addition, glycosylation at position 461 of loop V5 also made antibody binding more difficult (Fig 5F). Reverting Asn461 to Ala made CNE23, CNE63 and TV1.29 sensitive to VRC01 class antibodies. However, removing the glycan at position 461 reduced the sensitivity to ibalizumab.

Unlike the VRC01 class antibodies, VRC13 and VRC16 primarily use their CDRH3 domains to bind HIV-1 gp120[17] and were less sensitive to the signature substitutions, especially those mutations in CNE6, CNE63, CNE64 and TV1.29 (Table 2). A comparison of the antibody footprints on HIV-1 Env indicated that the epitope of VRC13 is slightly distant from loop D and loop V5, and shifted more towards the trimer apex despite significant overlap with the epitope of VRC01 class antibodies (Fig 5B). Consequently, several critical contact residues for VRC01 class antibodies, such as the loop D residues Asn279 and loop V5 residues Gly459 and Asn461, no longer interact with VRC13.Even though the main-chain O atom of loop D residue Ala281 makes a hydrogen bound with VRC13 residue Asn100_G_ along the side of CDR H3, mutation to other residues with bulkier side chains did not create any steric clashes with VRC13. Therefore, mutations in CNE6, CNE63, CNE64 and TV1.29 did not confer resistance to VRC13. By contrast, Gly471 of the outer domain exiting loop residue lies underneath the CDR H3 of VRC13 and makes a hydrogen bond to Arg98 in CHR H3 with its main-chain O atom. Modeling indicated that any mutations from a glycine at position 471 would result in steric clashes with VRC13 and thus confer resistance (Fig 5D). This was consistent with the observation that CNE23 and CNE66 were resistant to VRC13 and reverting Glu471 to Gly essentially restored their sensitivity(Table 2). Therefore, even though VRC01 class antibodies and VRC13 target the same general area on the CD4bs, a slight difference of gp120 recognition gives them different sensitivity to resistance mutations on the CD4bs.

Collectively, signature mutations that confer resistance to the CD4bs antibodies were located on multiple regions within the CD4bs supersite. Mechanistically, those mutations could act by abolishing hydrogen bonds and/or salt bridges, as shown for mutations at position 97 of gp120 to non-lysine residues and at position 279 to non-Asn/Asp residues. Mutations could also act by imposing steric hindrance to antibody contact, as observed in mutations to amino acids with bulkier side chains from Ala at position 281, Thr at position 455 and Gly at position 471. Altering a potential glycosylation site is another mode of resistance as observed in many resistant strains with glycosylation at position 461.It is of note that the VRC01 class antibodies, as well as the non-VRC01 class antibodies such as VRC13, which use the canonical CDR H3 for recognition, could tolerate different resistance mutations on HIV-1 gp120, potentially due to their distinct binding modes [17].

## Discussion

A new generation of bnAbs has emerged as promising candidates for clinical intervention against HIV-1 infection.However, the existence and emergence of resistance would impose substantial challenges for optimal clinical outcomes. In this study, we identified and characterized diverse HIV-1 clones with resistance to a large number of bnAbs, particularly those targeting the CD4bs (Resistant panel to CD4bs bnAbs). These clones represent a small but significant proportion of a large envelope that we collected from acutely and chronically infected patients, as well as from literature reports[39, 48–50]. Importantly, all of these clones were isolated from patients with diverse ethnic and geographic origins and during various stages of disease progression and transmission. Site-directed mutagenesis and structural analysis revealed key residues within the bnAbs epitopes and the molecular features that confer resistance. Resistance is largely correlated with reduced binding avidity of antibodies to the quaternary trimeric envelope protein expressed on the cell surface, although steric hindrance by bulky side-chains and glycans proximate to the epitope was also detected.These findings are in agreement with the observational evidence that there is naturally occurring resistance against CD4bs antibodies.Treatment strategies utilizing these antibodies would need to overcome such resistance to achieve optimal clinical outcomes.

Several findings of our study are worth highlighting here. First, HIV-1 strains with resistance to CD4bs bnAbs appear to be more prevalent than expected. Among the full-length clones that we previously characterized, approximately 12% were found to be broadly resistant, possibly because many clones were isolated during the chronic stage of infection, when a more divergent swarm of HIV-1 strains is present than in acute and early stages[56–60]. To some extent, this result is reminiscent of findings from human clinical trials in which bnAb monotherapy regimens were evaluated in chronic patients, whereby less sensitive and resistant HIV-1 strains were frequently detected in the rebounding viral populations [11, 15, 43, 46, 47].However, this by no means indicates that broadly resistant strains cannot arise during the acute or early phase of infection. In fact, among the 19 broadly resistant strains highlighted here, 2 came from acute and 8 from early infected patients (Table 1). Although their prevalence remains uncertain, their existence implies that they can successfully compete with other wild-type clones and be transmitted to uninfected individuals. In this regard, combination therapy with bnAbs targeting more than one vulnerable site on the envelope would be a more efficacious approach than monotherapy [1, 9, 10, 61]. Similar lessons have already been learned in the development of antiretroviral drugs, where combination therapyis required for protection against resistance and to achieve sustained suppression of viral replication[62, 63].Ultimately, treatment strategies that integrate antiretroviral drugs and bnAbs are more likely to maximize the potential of both classes.

**Fig 6:**
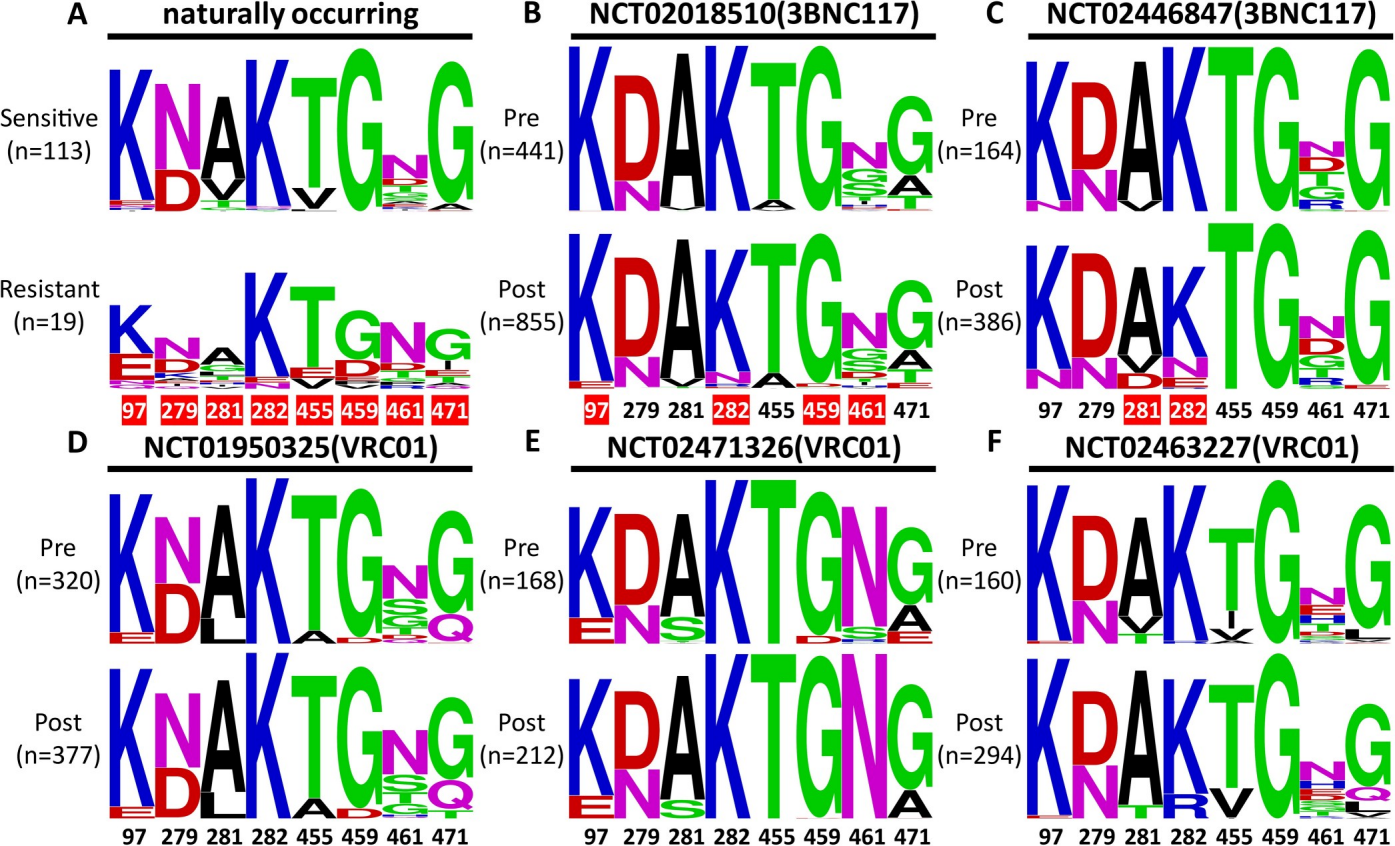
Polymorphisms and shifts at the signature residues before and after treatment with 3BNC117 and VRC01. The logograms show the frequency/height of each signature residue between **(A)** the sensitive strains retrieved from the HIV database and the broadly resistant HIV-1 strains highlighted in the current study, (**B**, **C**) pre- and post-treatment with 3BNC117, and (**D**,**E**, and **F**) pre- and post-treatment with VRC01. The trial registration numbers and the actual number of HIV-1 sequences retrieved and analyzed are indicated.Those that demonstrated substantial changes (> 10-fold or newly emerged) are indicated in red boxes. All numbers are based on the HIV-1 HXB2 sequence. The signature residues are colored according to their distinct biochemical properties as in Fig 1.

Secondly, we found that multiple substitutions at one or more contact regions targeted by the antibodies were required to fully restore the antibody-sensitive phenotype in most cases. The contribution of each residue to overall resistance varied for each antibody, highlighting the complex nature of immune system evasion by HIV-1 as it emerges and spreads. In this regard, the broadly resistant strains were more likely the product of continued viral evolution under immune pressures,rather than a single instantaneous event. In fact, some of the key contact site residues responsible for broad resistance were frequently shared among the less sensitive HIV-1 strains that were identified from natural infections, as well as in animal models and bnAb monotherapy human trials[15, 43–45, 47, 64]. For example,nearly all HIV-1 variants found in recent plasma samples from the original VRC01 donor are now resistant to VRC01 [42], as are the VRC01-resistant strains we previously identified in a chronically infected patient[39–41].Similar to what we found in the current study, the mutations responsible for such resistance are also confined to the area between residues 278 and 283 of Loop D, and to potential N-linked glycosylation sites between residues 458 and 467 of the β23/loop V5/β24 region. Analyses of rebound viruses in humanized mice or rhesus macaque models after antibody monotherapy with 3BNC117 and N6 also revealed mutations between 279–281 of loop D, and between 458–459 of the β23/loop V5/β24 region [44, 45] In particular,human monotherapy trials with the 3BNC117 and VRC01 antibodies revealed significant polymorphisms. A shift in HIV-1 plasma RNA populations was found at the abovementioned positions (Fig 6).Due to pre-selection for 3BNC117-sensitive participants, the shift was more profound in 3BNC117 than in the VRC01 trial[11, 15, 43, 46, 47]. Most noticeably, there was a more than 10-fold increase in the frequency of glutamic acid (E) at position 97, possibly to disrupt the salt bridges between Lys97 and an aspartic acid in CDR H3 of VRC01 class antibodies, as well as in aspartic acid (D) at position 459, after treatment with 3BNC117 (Fig 6B). The emergence of an asparagine residue (N) at position 461was also found after treatment (Fig 6B).Similarly, the emergence of aspartic acid (D) at position 281 and glutamic acid (E) and asparagine (N) at position 282 was also identified in the second 3BNC117 trial (Fig 6C).However, the shift of residue frequencies was quite minimal in the VRC01 trials[11, 46], although the high degree of polymorphism will undoubtedly provide a rich genetic base for future selection (Fig 6D–6F). Collectively, these results suggest that the broadly resistant HIV-1 strains identified here arose under selection pressure from CD4bs-directed immune response during both natural infection and CD4bs bnAbs treatment.

Finally, as none of the Chinese patients and highly unlikely the non-Chinese patients studied here developed bnAbs, the resistant viruses identified here are unlikely to have resulted from selection forces equivalent to the bnAbs being studied here. What exactly is being selected for and how these mutations emerged in the first place therefore remains unclear. We believe that the broadly resistant strains are naturally occurring, and their emergence is likely the result of a combination of random mutational events due to the error-prone nature of HIV-1 reverse transcriptase and immune selection for viral escape. Major mechanisms appear to include a reduction of antibody binding to the contact residues and increased steric obstruction imposed by the bulky side-chains or glycansat the mutated residues.As the HIV-1 pandemic continues to evolve and particularly more CD4bs antibodies are entering clinical studies, more resistant strains are expected to arise. To overcome this resistance for optimal clinical outcomes, more potent and broadly effective antibodies are needed, either by isolating antibodies with exceptional neutralizing activity from patients,or engineering existing antibodies into bi- or tri-specific combinations [7, 8, 27, 28, 65–68].A combination of VRC01 class and CDR H3-dominated antibodies, such as VRC13, may also be a beneficial alternative for developing therapeutic antibody cocktails due to their different sensitivity to resistance mutations at the CD4bs.

## Materials and methods

### Envelope clones, antibodies and soluble CD4

The envelope clones CNE6, CNE23, CNE63, CNE64 and CNE66 were isolated from HIV-1 infected patients as previously reported [39]. BL01, TV1.29 and TZA125.17 were or obtained from Dr. John Mascola at the Vaccine Research Center of the NIH, USA, and BJOX2000 from Dr. Feng Gao at Jilin University, China.A total of 30 HIV-1 antibodies recognizing the CD4 binding site (CD4bs), the V1V2 apex, the V3 glycan, the CD4 induced (CD4i), the subunit interface, the fusion peptide (FP) and the membrane proximal external region (MPER) of gp41 were used to evaluate the neutralization sensitivity of the pseudoviruses bearing the abovementioned envelope clones. The antibodies were kindly provided by Drs. John Mascola of the Vaccine Research Center at NIH, Michel Nussenzweig at Rockefeller University, Barton Haynes at Duke University, Dennis Burton at the Scripps Research Institute, and Wayne Koff at the Human Vaccine Project. For some antibodies with crystal structures and published sequences, the variable genes were synthesized and cloned into the human IgG1 expression vectors provided by Michel Nussenzweig at Rockefeller University[37]. The full-length IgG1 was expressed by co-transfecting HEK293F cells (ATCC) with paired heavy- and light-chain plasmids and purified by affinity chromatography using Protein A columns (Thermo Scientific), following the manufacturer's instructions. The antibody concentration was determined using BCA Protein Assay Kit (Thermo Scientific). Ibalizumab, an antibody against the second cellular domain of the human CD4 molecule, was kindly provided by Dr. David Ho at the Aaron Diamond AIDS Research Center of Rockefeller University. Soluble CD4 encoding the first two domains linked to the Fc was expressed, purified and quantified as described above for the antibodies.

### Mutant envelopes, pseudovirus production and neutralization assay

A total of 58 mutant envelope clones of CNE6, CNE23, CNE63, CNE64, CNE66, BJOX2000, BL01, TV1.29 and TZA125.17 were generated using a site-directed mutagenesis kit (Agilent) and confirmed by sequencing. The mutant envelopes included single, double, tripleand quadruple substitutions, as well as various possible combinations thereof. The mutant JRFL envelopes containing multiple substitutions derived from CNE6, CNE23, CNE63, CNE64, CNE66 and BJOX2000 were also generated and confirmed by sequencing. Pseudoviruses bearing the wild-type and mutant envelopes were generated by co-transfecting HEK293T cells with Env expression vectors and the pNL4–3R-Eluciferase viral backbone plasmid as described previously [69]. Pseudovirus-containing supernatants were collected 48 hours post transfection and the viral titers were measured by luciferase activity in relative light units (RLU) (Bright-Glo Luciferase Assay System, Promega Biosciences, California, USA).The supernatants were aliquoted and stored at -80°C until further use. Neutralization assays were performed by adding 100 TCID50 (median tissue culture infectious dose) of pseudovirus into 8 serial 1:3 dilutions of purified antibody starting from 50 μg/ml. The mixture was then dispensed into a 96-well plate in triplicate and incubated for 1 h at 37°C.Approximately 1.5x10^4^ GhostX4/R5 cells were then added and the cultures were maintained at 37°C for an additional 72h before luciferase activity was measured.Neutralizing activity was measured by the reduction in luciferase activity compared to the controls. Fifty- and eighty percentmaximal inhibitory concentrations (IC_50_ and IC_80_), the concentrations required to inhibit infection by 50 and 80% compared to the controls, were calculated using the dose-response-inhibition model with 5-parameter Hill slope equation in GraphPad Prism 7(GraphPad Software, USA.). The IC_50_ and IC_80_ values for the reported viruses (T278-50, 242–14, T250-4, HO86.8, DU422.01, X2088.c9, 6322.V4.C1, 6471.V1.C16, 6631.V3.C10, 620345.c1) were obtained from CATANP (https://www.hiv.lanl.gov/components/sequence/HIV/neutralization/)[70].

### Sequence alignments and consensus sequence logo

The gp160 protein sequences of resistant and sensitive HIV-1 strains were downloaded from the Los Alamos HIV sequence database (www.hiv.lanl.gov) and aligned against the subtype B reference sequences HXB2, SF162 and JRFL using BioEdit (http://www.mbio.ncsu.edu/bioedit/bioedit.html). The frequencies of different residues at positions 90–100, 275–283 and 455–476 was analyzed and sequence logos were generated using the WebLogo 3.0 tool (http://weblogo.threeplusone.com/) [51].

#### Antibody binding to the envelope trimers on the cell surface

A total of 4 × 10^6^ HEK 293T cells were seeded into 10-cm round cell culture dishes and incubated at 37°C. After 24h, the cells were transfected with a total of 6μg of Env expression plasmid mixed with 24μg PEI in 1ml Opti-MEM (Gibco, USA) and incubated for 36 hours at 37°C. The cells were harvested and distributed into 96-well round-bottom tissue culture plates for the individual staining reactions. For each staining reaction, cells were washed thrice with 200μl staining buffer (PBS with 2% heat-inactivated FBS (Gibco, USA) and 2 mM EDTA). The cells were stained for 45 minutes in 50μl of staining buffer with 10μg/ml of primary antibody at room temperature. After washing thrice with 200μl of staining buffer, the cells were stained with PE labeled anti-human IgG secondary antibody (rabbit anti-human IgG-PE, Santa Cruz) at a 1:200 dilution in 50μl of staining buffer for 45 minutes at room temperature. Following three washes with staining buffer, the cells were resuspended and analyzed on a FACSCalibur instrument (BD Biosciences, USA) using FlowJo 10 software (FlowJo, USA) [71]. Appropriate negative controls were included, such as mock-transfected 293T cells, secondary conjugated antibody labeling of Env-transduced cells as a background control for the secondary antibody, and the irrelevant antibody MERS-4V2 targeting the receptor binding domain of Middle East Respiratory Syndrome Coronavirus[72]. The antibody 10–1074 recognizing the glycan-V3 loop was used as a primary positive control[73].

### Assay for cell entry dependent or independent on CD4

The Cf2Th.CD4.CCR5 cell line expressing both human CD4 and CCR5 and the Cf2Th.CCR5 cell line expressing only human CCR5 were used to evaluate the dependence of viral entry on CD4 molecules.They were kindly provided by Dr. Cecilia Cheng-Mayer at the Aaron Diamond AIDS Research Center of Rockefeller University. Briefly, 100 μl of each undiluted viral stock was incubated with 20 μl of medium containing 1x10^4^ Cf2Th.CD4.CCR5 or Cf2Th.CCR5 cells at 37°C. The culture was fed with 80μl fresh complete medium on the following day. Virus entry was measured 1 day later by determining the luciferase activity in the cell lysates in relative light units (RLU) (Bright-Glo Luciferase Assay System, Promega Biosciences, California, USA)[42, 74, 75].

### Structural analysis

HIV-1 Env structures in complex with VRC01 (5FYJ), 3BNC117 (5V8M) and structures of HIV-1 core gp120 in complex with antibodies N6 (5TE6), 8ANC131 (4RWY), VRC13 (4YDJ) and VRC16 (4YDK) were superposed over the outer domain regions (residues 252:476). Mutations were modeled using the mutagenesis function of PyMOL. Antibody-antigen interactions, including hydrogen bonds and salt bridges, were identified using PDBePISA (http://www.ebi.ac.uk/msd-srv/prot_int/pistart.html).

### Statistical analysis

Geometric mean fluorescence intensity (GMFI) was calculated using FlowJo 10 software (FlowJo, USA). Pearson correlation coefficients and *P*-values of differences between GMFI and IC_50_/IC_80_ for each antibody were calculated using GraphPad Prism 7 (GraphPad Software, USA).

## Supporting information

S1 Fig Binding activity of bnAbs to wild-type and mutant Env trimers expressed on the surface of HEK293T cells analyzed by flow cytometry. Each broadly resistant strain and their mutated clones are separately presented in (A) for CNE6, (B) for CNE23, (C) for CNE63, (D) for CNE64, (E) for CNE66, and (F) for BJOX2000. The actual residue substitutions for each clone are indicated at the top of each graph. Numbers in the gates represent the geometric fluorescence intensity (GMFI) from one experiment. The negative controls include mock-transfected HEK293T cells for background labeling by bnAbs, secondary conjugated antibody labeling of Env-transduced cells for the background control of the secondary antibody, and the irrelevant antibody MERS-4V2 targeting the receptor binding domain of Middle East Respiratory Syndrome Coronavirus. Antibody 10–1074 recognizing the glycan-V3 loop was used as a primary positive control.
(PDF)
