# Supplementary figures and images for: Broadly resistant HIV-1 against CD4-binding site neutralizing antibodies
Source: PLoS Pathog. 2019 Jun 13;15(6):e1007819. doi: 10.1371/journal.ppat.1007819 (PMC6592578; doi:10.1371/journal.ppat.1007819)

A

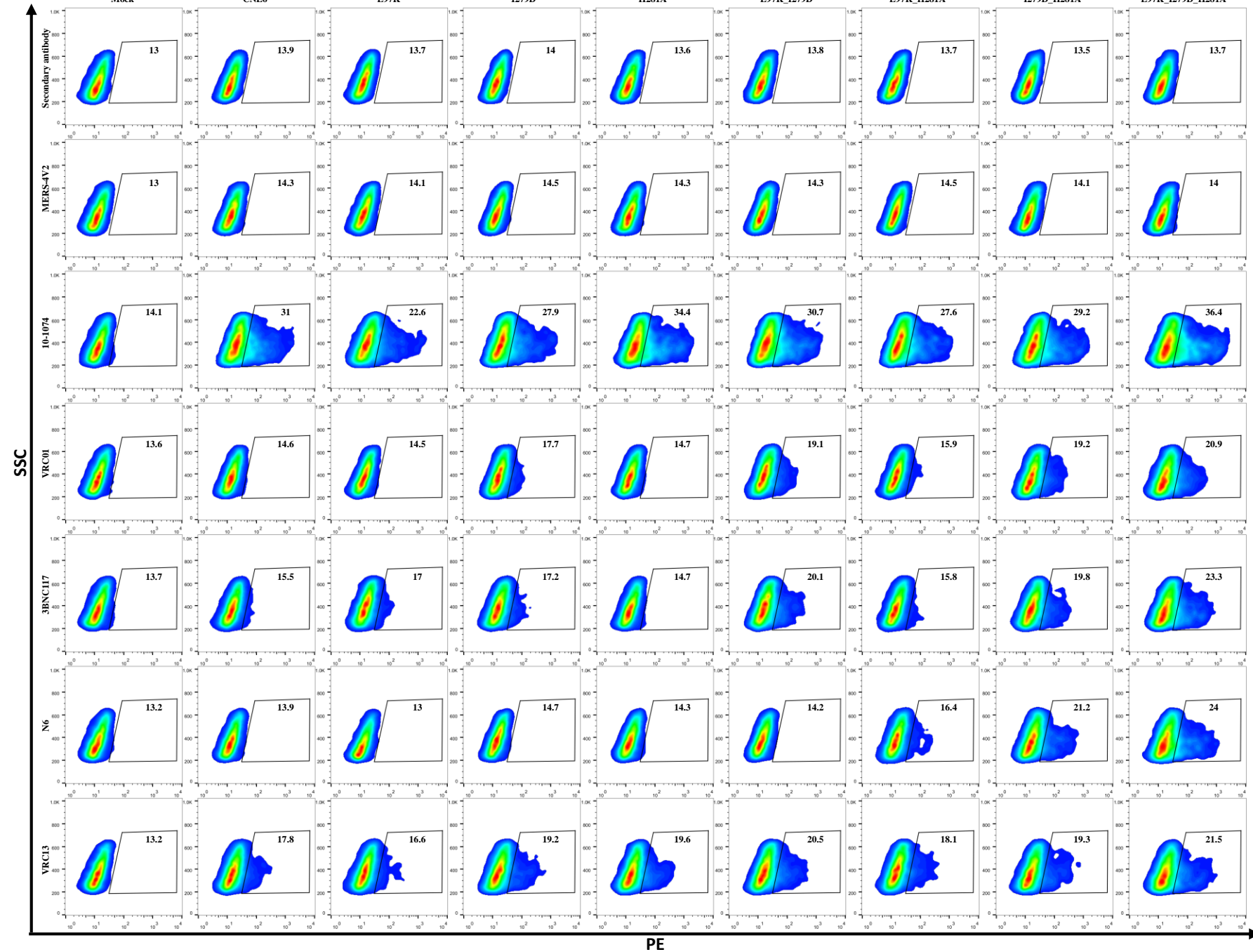

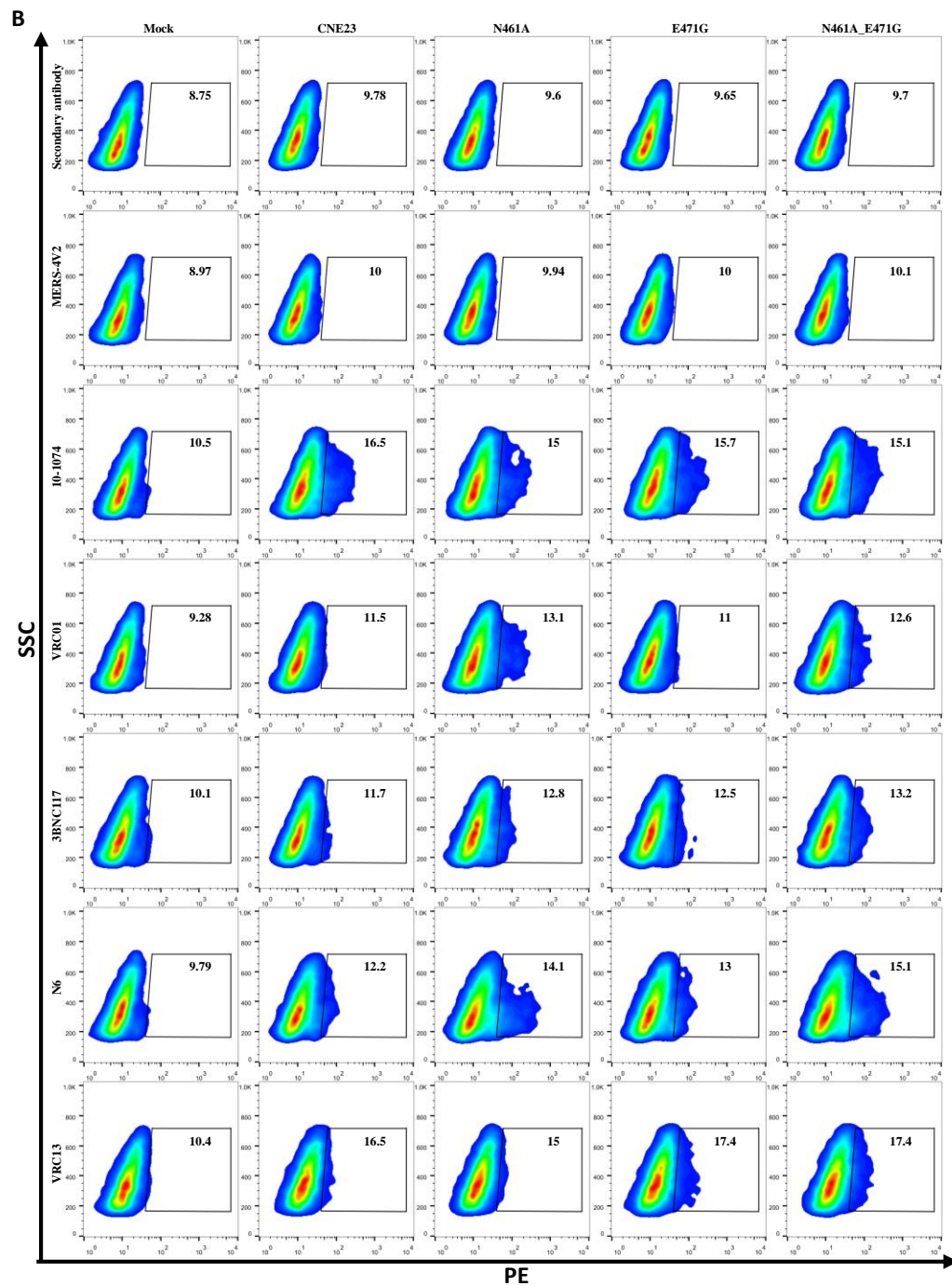

C

SSC

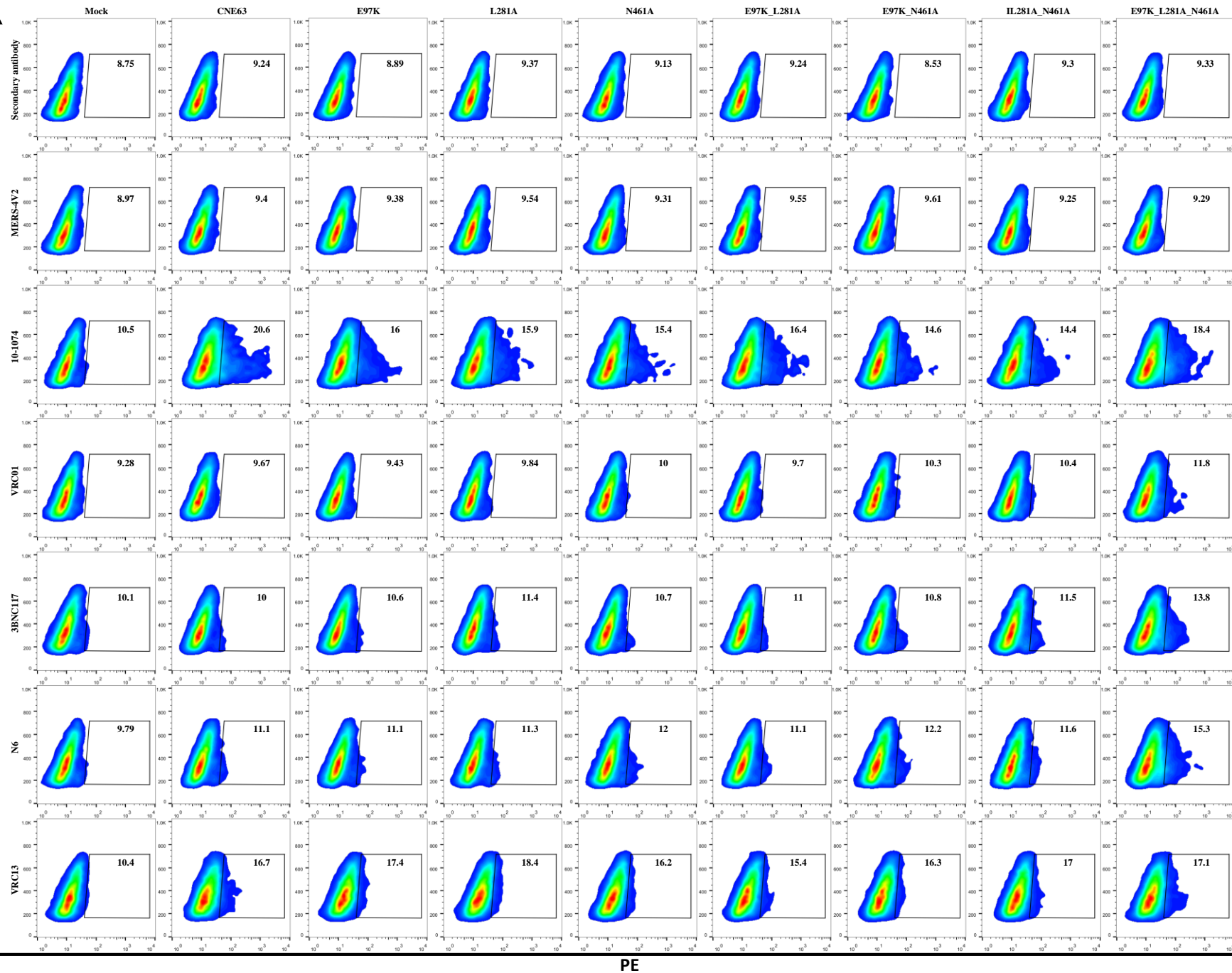

PE

D

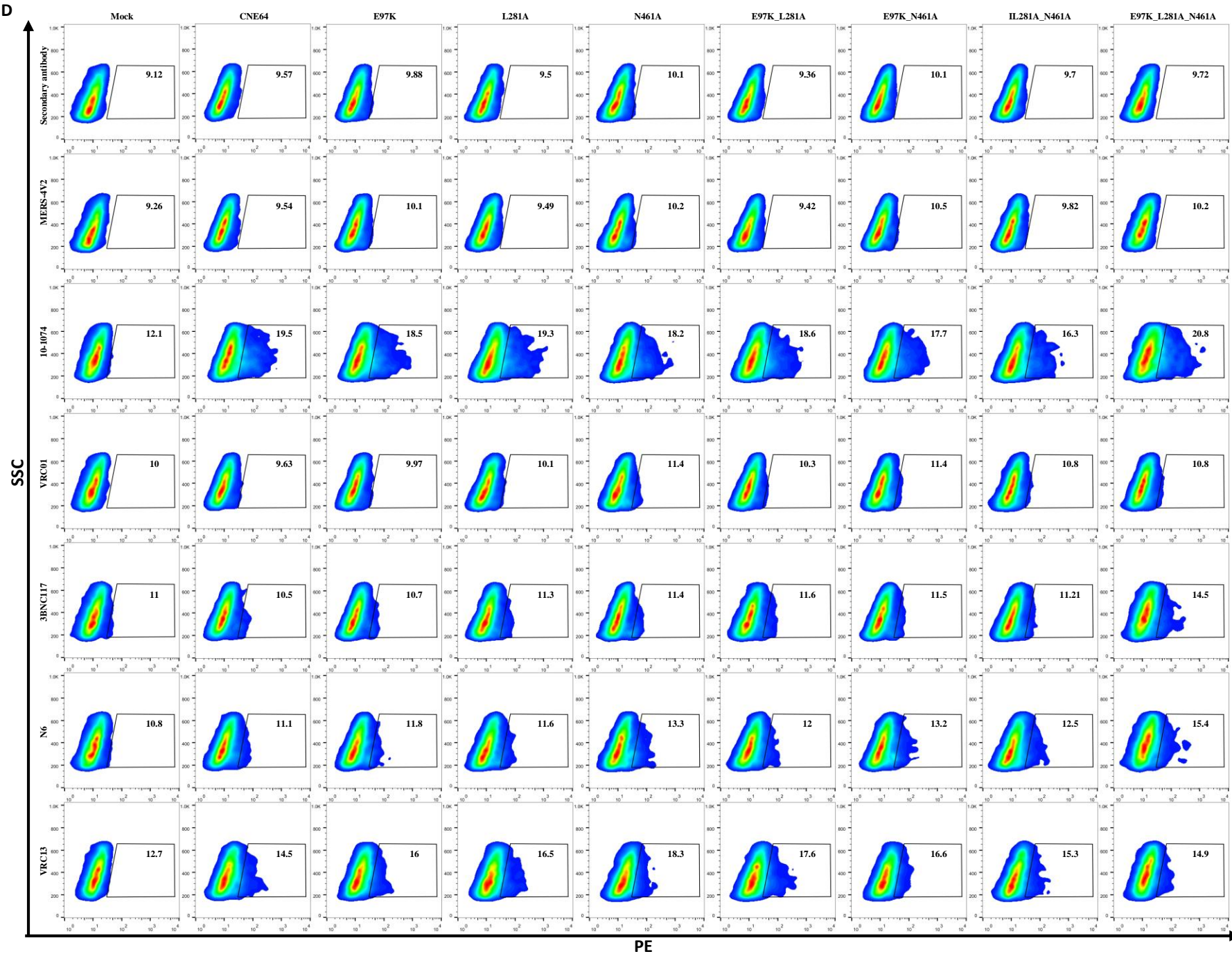

E

SSC

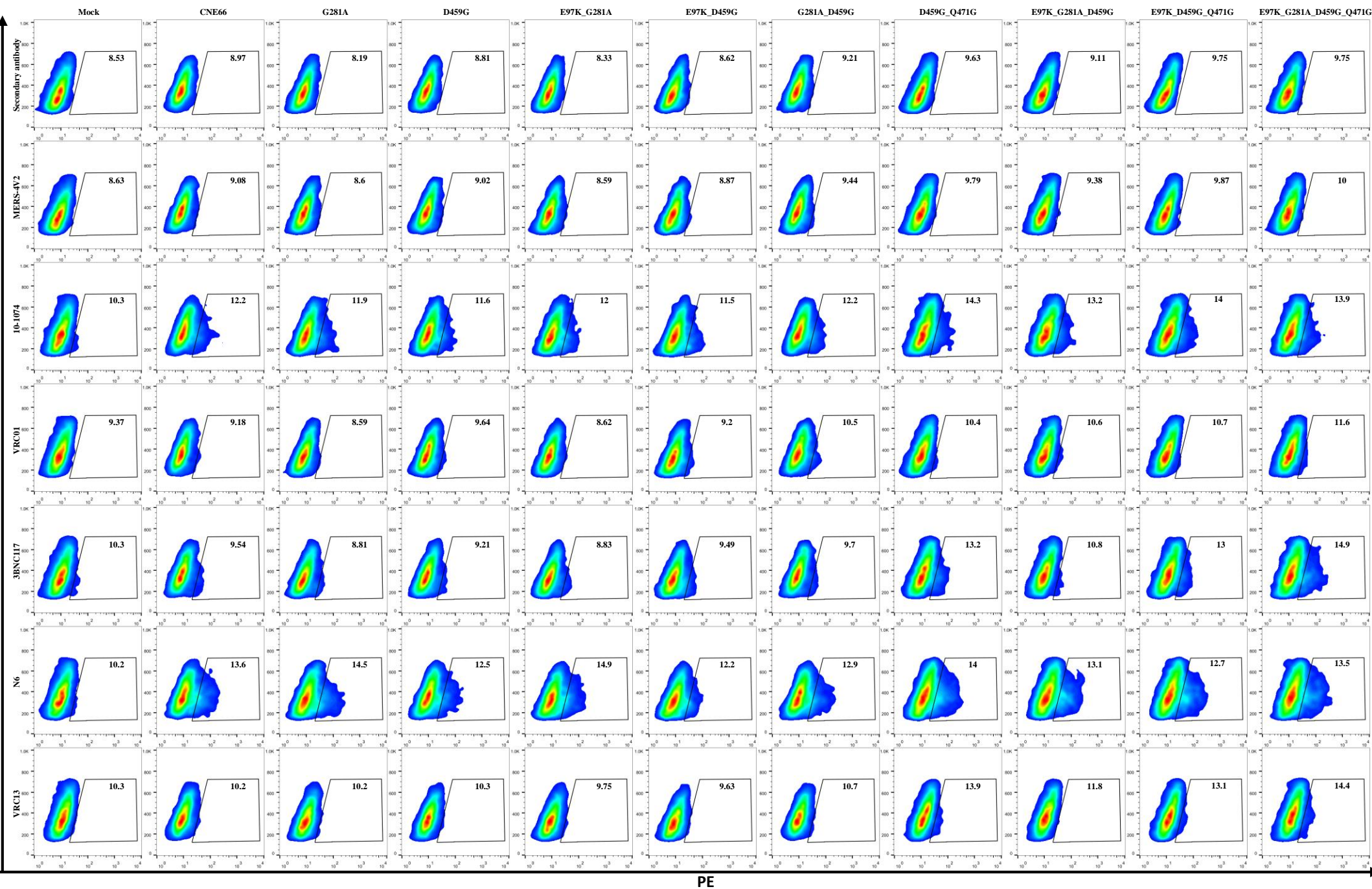

PE

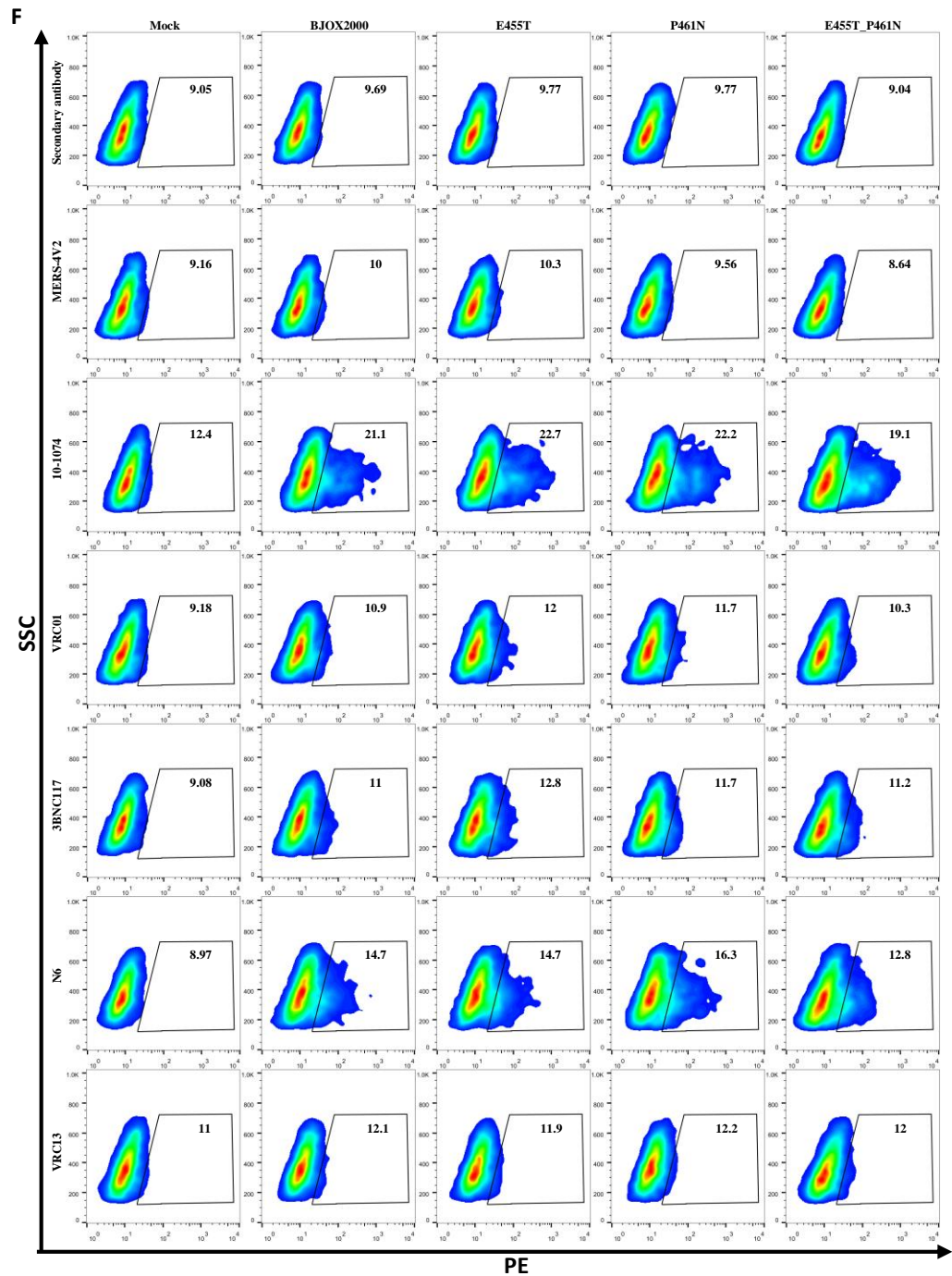

Supplement: S1 Fig — Each broadly resistant strain and their mutated clones are separately presented in (A) for CNE6, (B) for CNE23, (C) for CNE63, (D) for CNE64, (E) for CNE66, and (F) for BJOX2000. The actual residue substitutions for each clone are indicated at the top of each graph. Numbers in the gates represent the geometric fluorescence intensity (GMFI) from one experiment. The negative controls include mock-transfected HEK293T cells for background labeling by bnAbs, secondary conjugated antibody labeling of Env-transduced cells for the background control of the secondary antibody, and the irrelevant antibody MERS-4V2 targeting the receptor binding domain of Middle East Respiratory Syndrome Coronavirus. Antibody 10–1074 recognizing the glycan-V3 loop was used as a primary positive control. (PDF) [file ppat.1007819.s001.pdf]
